# Image-guided focused ultrasound for non-invasive modulation of superficial and deep tissues

**DOI:** 10.1016/j.xpro.2026.104900

**Published:** 2026-10-19

**Authors:** Namrata Sangwan, N. Chris Chaggares, Zahra Torbatian, Thomas Anthony Anderson

**Affiliations:** 1 Department of Anesthesiology, Perioperative and Pain Medicine, School of Medicine, Stanford University, Stanford, CA, USA; 2 SONELE Inc., Markham, ON, Canada

**Keywords:** Health Sciences, Neuroscience, Systems biology

## Abstract

Focused ultrasound (FUS) enables non-invasive, spatially precise modulation of biological tissues. This protocol describes assembly, calibration, and implementation of a diagnostic ultrasound-guided FUS (dUS-FUS) platform for real-time targeting of superficial and deep tissues in small-animal models. We demonstrate reversible sciatic nerve modulation in a rat acute pain model and assess sensory and motor functions using behavioral tests. The platform provides a reproducible workflow for image-guided tissue modulation and can be adapted to diverse preclinical applications.

For complete details on the use and execution of this protocol, please refer to Sangwan et al.^1^^,^^2^

## Highlights

• Steps for assembly, calibration, and dUS-guided focused ultrasound targeting
• Instructions for non-invasive modulation of superficial and deep tissues
• Guidance on reversible sciatic nerve modulation and behavioral assessment
• Protocol adaptable for neuromodulation, tissue ablation, and targeted delivery

## Abstract (editor highlights)

Publisher’s note: Undertaking any experimental protocol requires adherence to local institutional guidelines for laboratory safety and ethics.

## Graphical abstract

**Figure:**
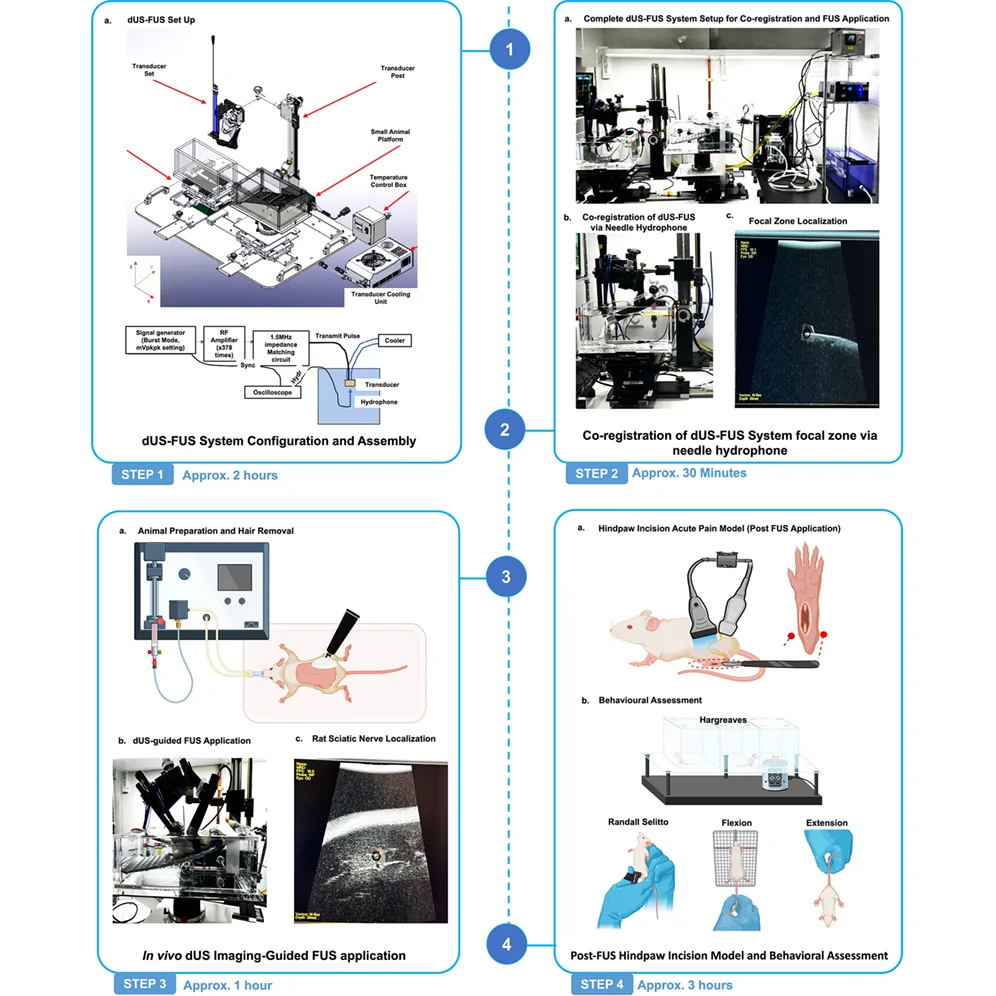

## Before you begin

The ability to precisely and non-invasively manipulate biological tissues *in vivo*, with high spatial specificity and without pharmacological confounds, has become an essential demand in experimental biology. Focused ultrasound (FUS), particularly when integrated with imaging guidance, represents a powerful convergence of physics, engineering, and biology that meets this need.^3^^,^^4^ Unlike traditional approaches such as surgery, implanted devices, or systemic drug delivery, which suffer from limitations including invasiveness, off-target effects, and limited spatial specificity, FUS offers a non-invasive, rapidly tunable, and highly focal approach to modulate, ablate, or sensitize tissues across organ systems.^5^^,^^6^

Clinical translation of magnetic resonance imaging (MRI)-guided FUS has accelerated, with FDA-approved applications for thermal ablation in essential tremor and tremor-dominant Parkinson’s disease and ongoing investigation in epilepsy, obsessive-compulsive disorder, major depressive disorder, and chronic pain.^7^^,^^8^^,^^9^^,^^10^ Beyond ablation, FUS is being investigated for blood–brain barrier (BBB) opening, neuromodulation, targeted drug delivery, and immune modulation within the central nervous system.^6^^,^^11^ Early human studies of BBB opening in Alzheimer’s disease have demonstrated safety and feasibility, supporting further investigation with biologics, gene therapies, and nanoparticles.^12^ However, MRI-guided FUS requires specialized infrastructure and lengthy imaging procedures, limiting its accessibility for longitudinal and exploratory small-animal studies.^5^^,^^6^ For many preclinical applications, diagnostic ultrasound (dUS) provides a practical alternative through portable, real-time imaging and dynamic tissue targeting.^13^^,^^14^

FUS applications extend well beyond the brain; *in vivo* studies have demonstrated its use for reversible inhibition of peripheral nerve conduction while preserving structural integrity and function,^15^^,^^16^^,^^17^^,^^18^ thermal ablation of tumors, including liver and prostate models,^19^^,^^20^^,^^21^^,^^22^ transient disruption of vascular barriers to facilitate drug uptake,^23^^,^^24^ as well as non-invasive modulation of neural circuits in the brain.^25^^,^^26^ Many of these effects have traditionally required surgical or invasive procedures, implanted devices, or systemic pharmacological interventions, which can introduce biological variability and additional experimental complexity.^27^^,^^28^^,^^29^^,^^30^^,^^31^^,^^32^ Similarly, modulation of vascular permeability, immune signaling, and cellular activity has often relied on systemic therapies associated with off-target effects, limited tissue specificity, and variable biodistribution.^31^^,^^33^^,^^34^ In contrast, FUS can produce spatially localized biological effects non-invasively through thermal and mechanical mechanisms, including cavitation, acoustic radiation force, acoustic streaming, and mechanotransduction, while minimizing disruption to surrounding tissues.^33^^,^^35^ Although image-guided approaches such as CT- or MRI-navigation provide high anatomical fidelity and real-time tissue temperature measurements, they are less suited for dynamic, real-time tissue targeting.^6^ dUS, in contrast, is portable, more affordable, and provides real-time visualization of tissues, making it particularly suitable for acute and longitudinal studies in small-animal models.^5^^,^^13^^,^^14^ Collectively, these features position dUS-guided FUS as a practical and scalable platform for real-time, image-guided tissue modulation in preclinical research, with applications ranging from non-invasive tissue ablation to targeted neuromodulation, and other tissue-specific applications that will require target-specific validation.

### Innovation

Methods for modulating superficial and deep tissues in small-animal models often rely on invasive surgery, systemic pharmacological agents, implanted devices, or genetic tools, all of which may limit spatial precision, reproducibility, and translational relevance. To address these limitations, we developed a noninvasive diagnostic ultrasound-guided focused ultrasound (dUS-FUS) platform for peripheral nerve modulation that enables real-time, image-guided, and spatially precise delivery of acoustic energy to biological tissues *in vivo*^5^^,^^6^ Although this protocol is validated for reversible sciatic nerve modulation in a rat acute pain model, the platform can be readily adapted to target a wide range of superficial and deep tissues.

First, this protocol integrates diagnostic ultrasound and focused ultrasound into a single platform, enabling simultaneous visualization, anatomical targeting, and delivery of acoustic energy during sonication. Unlike MRI-guided focused ultrasound systems, which require specialized infrastructure and prolonged imaging sessions, this approach is portable, bench-compatible, and readily implemented in routine preclinical laboratories.^5^^,^^6^

Second, the protocol provides a standardized workflow for platform assembly, transducer co-alignment using a needle hydrophone, system calibration, image-guided targeting, and *in vivo* implementation, thereby improving reproducibility and facilitating adoption across laboratories.

Third, the platform enables noninvasive tissue modulation without surgical exposure, implanted hardware, viral vectors, or genetic modification. By adjusting acoustic exposure parameters, the same system can be adapted for reversible neuromodulation, modulation of vascular permeability, targeted therapeutic delivery, hyperthermia, and tissue ablation across multiple organ systems.^4^^,^^13^^,^^23^^,^^36^^,^^37^

Finally, the protocol is validated in a rat acute pain model, in which diagnostic ultrasound is used to localize the sciatic nerve and focused ultrasound is applied to achieve reversible peripheral nerve modulation. Using this workflow, we demonstrated reversible inhibition of sciatic nerve function with recovery to normal physiological function within 4–5 weeks, providing a flexible and reproducible framework that can be readily adapted for image-guided investigations of peripheral nerves, vasculature, and other superficial and deep organs.^1^^,^^2^ Overall, this platform provides a versatile and scalable approach for noninvasive image-guided tissue modulation and supports a broad range of preclinical studies in neuroscience, oncology, regenerative medicine, and targeted therapeutic delivery.

### Institutional permissions

All animal procedures described in this protocol were approved by the Stanford University Administrative Panel on Laboratory Animal Care (APLAC) and were conducted in accordance with institutional guidelines, the National Institutes of Health Guide for the Care and Use of Laboratory Animals, and all applicable national regulations. Adult male and female Sprague-Dawley rats were used in this study. Investigators performing this protocol should obtain approval from their respective institutional animal care and use committee (IACUC) or equivalent regulatory authority before initiating animal experiments.

### Preparatory steps

1. Assemble the complete dUS-FUS platform, including the hydrophone calibration platform, small-animal platform, diagnostic ultrasound system, focused ultrasound system, positioning stages, and cooling system.
2. Prepare freshly degassed Milli-Q water and fill the hydrophone and small-animal water tanks.
3. Verify proper operation of the diagnostic ultrasound, focused ultrasound, RF amplifier, function generator, oscilloscope, hydrophone, and temperature control systems.
4. Prepare the anesthesia system, heating system, behavioral testing equipment, and sterile surgical instruments.
5. Perform transducer co-alignment and acoustic calibration before initiating image-guided focused ultrasound experiments.
6. Prepare animals according to institutional guidelines, including acclimation, hair removal, and baseline behavioral assessments.

## Key resources table

**Table:** 

REAGENT or RESOURCE	SOURCE	IDENTIFIER
**Experimental models: organisms/strains**		

Sprague Dawley rats (male and female, 200–250 g)	Charles River Laboratories	N/A

**Chemicals, peptides, and recombinant proteins**		

Isoflurane (Isospire™)	Dechra	Cat. no. 17033-091-25
Triple Antibiotic Ointment	We Care™	Cat. no. 1185
Systane Eye Ointment	Alcon	DIN02444062
Alcohol Swabs (Kendall Webcol Alcohol Preps)	Tyco Healthcare	Cat. no. 6818
Povidone Iodine	Up&Up™	#67777-141-50
Hair Removal Cream (Veet Sensitive Formula)	Veet	3116876
Filtered and degassed Milli-Q water	Milli-Q	Cat. no. ZIQ7000T0C
Sterile Cotton Gauze Pads	Fisherbrand	Cat. no. 22-415-468
Cotton-Tipped Wood Applicators	Dynarex Corporation	Cat. no. 4305
Disposable Sterile Towel Drapes	Dynarex Corporation	Cat. no. 4410

**Other**		

Focused Ultrasound (FUS) Transducer (1.5 MHz)	SONELE Inc., Markham, ON, Canada	Custom-built single-element 1.5-MHz FUS transducer; 16.5-mm focal depth; see Figure 2.2
Diagnostic Ultrasound (dUS) Imaging System (50 MHz)	Capistrano Lab, San Clemente, CA, USA	Custom 50-MHz dUS imaging system with UltraView XL software; see Figure 2.1
Needle Hydrophone (HNA 0400)	ONDA Corporation	HNA 0400
Hydrophone Tank	SONELE Inc.	Cat. no. PN 100309
Small Animal Tank	SONELE Inc.	Cat. no. PN 100563
Black Box Battery-Powered Control Module	SONELE Inc.	Cat. no. PN 92-017
Function Generator	Rigol	DG1022
Oscilloscope	Rigol	DS1054
RF Power Amplifier	E&I Pure Power	Model 2100L
Liquid Cooling System	Koolance	EXT-440
Coolant Liquid	Koolance	LIQ-705
Hydrophone Positioning Platform	Optics Focus	Custom-built; see Figures 4.1–4.6
Small Animal Positioning Platform	Optics Focus	Custom-built; see Figures 4.7–4.14
Temperature Controller	Tempco	TEC-9400
Temperature Logger	National Instruments (NI)	USB-TC01
Laptop Workstation for FUS Control	Dell Technologies	Precision 5550
Laptop Workstation for dUS Acquisition	Lenovo	N/A
UltraView XL Software	Capistrano Lab	N/A
VetFlo™ Traditional Anesthesia System	Kent Scientific Corporation	M-622642
Isoflurane Vaporizer	Patterson Veterinary	WV3IF06789
Feedback-Controlled Heating System (HP700 heating pad, REX-C100 PID controller, PT100 RTD sensor)	Kaz Inc.; Belme Intelligent Electric; T-Pro	HP700; REX-C100; PT100
Vacuum Tank	Terra Universal	Cat. no. 1255
Surgical Instruments (scalpel, blade, scissors, tissue forceps)	Various	N/A
Syringes (15/25 mL)	Various	N/A
Transducer Clamps	Optics Focus	Custom-built; see Figures 3.1 and 3.5
Oster Elite CryogenX Clipper (#50 Blade)	Oster Professional	N/A
Digital Weighing Balance	My Weigh	KD-8000
4-0 Nylon Monofilament Sutures	Cynamed	Cat. no. X001GR2159

**Software and Algorithms**		

UltraViewXL	CapoLabs	https://capolabs.com/
MATLAB	MathWorks	https://www.mathworks.com/products/matlab.html
Custom MATLAB code for function-generator control	This paper	Data S1
GraphPad Prism (version 10)	GraphPad Software	https://www.graphpad.com/

## Materials and equipment

The dUS-FUS platform consists of two independent water-tank systems designed for transducer calibration and *in vivo* image-guided focused ultrasound application (Figure 1). The hydrophone platform is used to perform transducer alignment and co-register the focal depths of the FUS and dUS transducers using a needle hydrophone. The small-animal platform is used for image-guided FUS application to the tissue of interest and incorporates a temperature-regulated water tank to maintain physiological conditions throughout the procedure (Figure 2.1).

**Figure 1:**
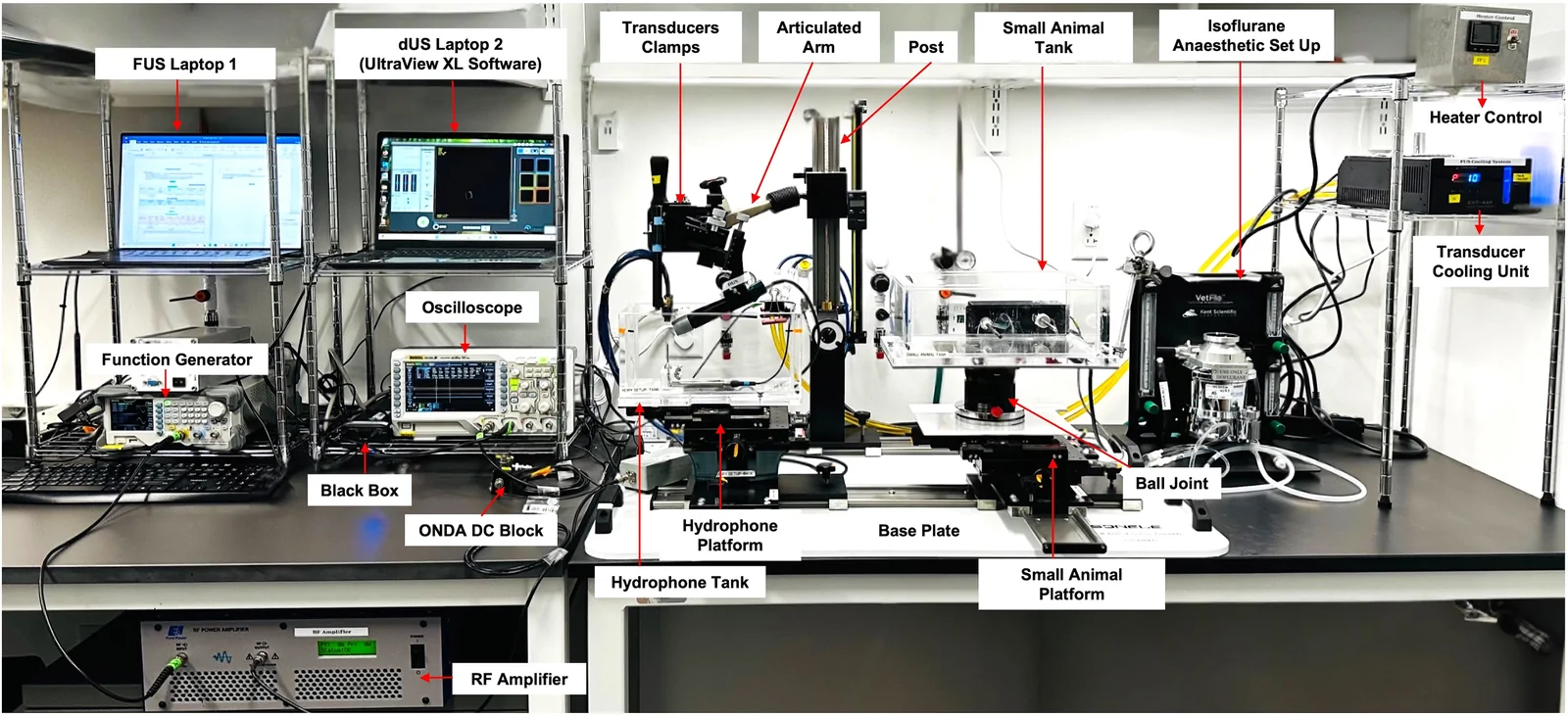
Overview of the Complete dUS-FUS System Setup Fully assembled diagnostic ultrasound-guided focused ultrasound (dUS-FUS) platform showing the small-animal experimental setup and the spatial arrangement and connections of the major system components used for image-guided FUS application (Preparatory Step 1).

**Figure 2:**
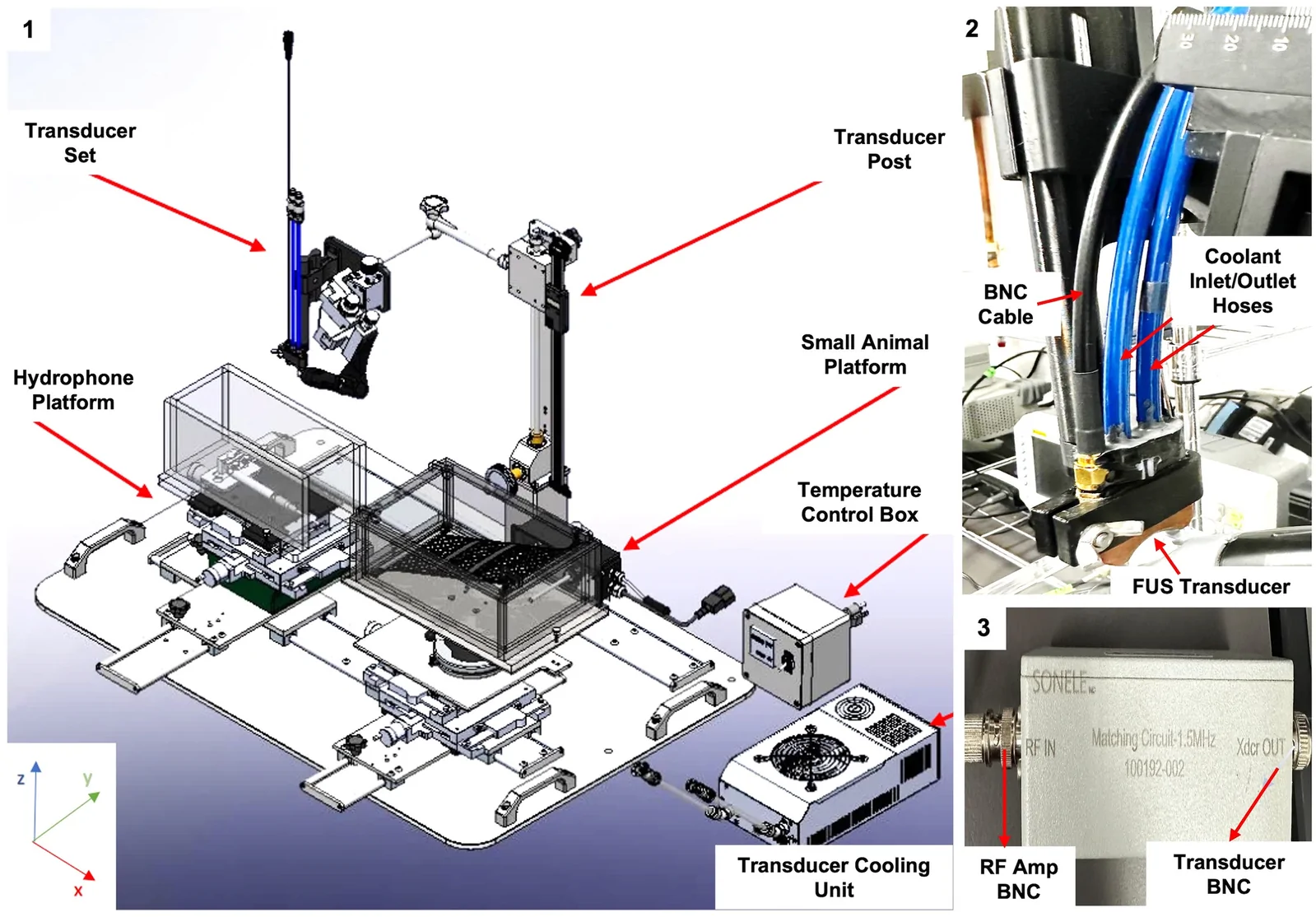
dUS-FUS System Set Up (1) Schematic illustration of the Small Animal System (Equipment Set Up, Steps 1–10). (2) Close-up view of the focused ultrasound (FUS) transducer (Equipment Set Up, Step 1a). (3) Illustration of the impedance matching circuit optimized for the FUS transducer (Equipment Set Up, Step 12b–c).

The FUS system consists of a single-element 1.5-MHz therapeutic transducer with a focal depth of 16.5 mm. The transducer is equipped with an impedance-matching circuit and an integrated liquid-cooling system to ensure stable acoustic output during sonication (Figures 2.2 and 2.3). The dUS system is used for real-time visualization of anatomical structures and guidance of FUS targeting.

Prepare filtered Milli-Q water by degassing it before each experiment using the vacuum tank. Use freshly degassed water as the acoustic coupling medium in both the hydrophone and small-animal water tanks to minimize acoustic attenuation and bubble formation.

The frequency response (Figure S1), acoustic output characterization (Figure S2), and pressure–voltage calibration (Figure S3) of the 1.5-MHz FUS transducer are provided as supplemental figures. Complete transducer co-alignment on the hydrophone platform before performing image-guided FUS experiments.

### Software

Four software and data-acquisition tools are used in this protocol.

#### UltraViewXL software

Used exclusively for dUS-based real-time imaging (with OPI LLC dUS transducers), enabling visualization and acquisition of tissue data during targeting (Figure 10.5). General information is available at https://capolabs.com/.

#### MATLAB

Used to control the function generator and the FUS transducer drive signal (setting the waveform, amplitude, and output of the function generator from the laptop workstation for FUS control). The custom MATLAB scripts are provided as a supplemental data file (Data S1; see Data and code availability). In this protocol, the alignment and *in vivo* settings are loaded from the function-generator preset files (FUS1.RSF and fusrat1.RSF; Steps 17a and 18e). General information is available at https://www.mathworks.com/products/matlab.html.

#### NI USB-TC01 temperature logger

Used for real-time temperature monitoring of the FUS transducer (thermocouple attached to the housing, measuring the temperature of the face of the transducer) during FUS application (Figures 10.5 and 11.7). The logger records transducer temperature only; it does not measure tissue temperature. General information is available from the NI USB-TC01 product page.

#### GraphPad Prism (version 10)

Used for statistical analysis (see quantification and statistical analysis). General information is available at https://www.graphpad.com/.

## Step-by-step method details

### Equipment setup

1. Assemble and position the FUS transducer.
  a. Attach the FUS transducer (Figure 3.1).
    i. Slide in the FUS transducer from the top.
    ii. Align the top of the transducer copper shell to the top of the clamp.
    iii. Rotate the wing nut clockwise to tighten the clamp until the transducer is fixed.

**Figure 3:**
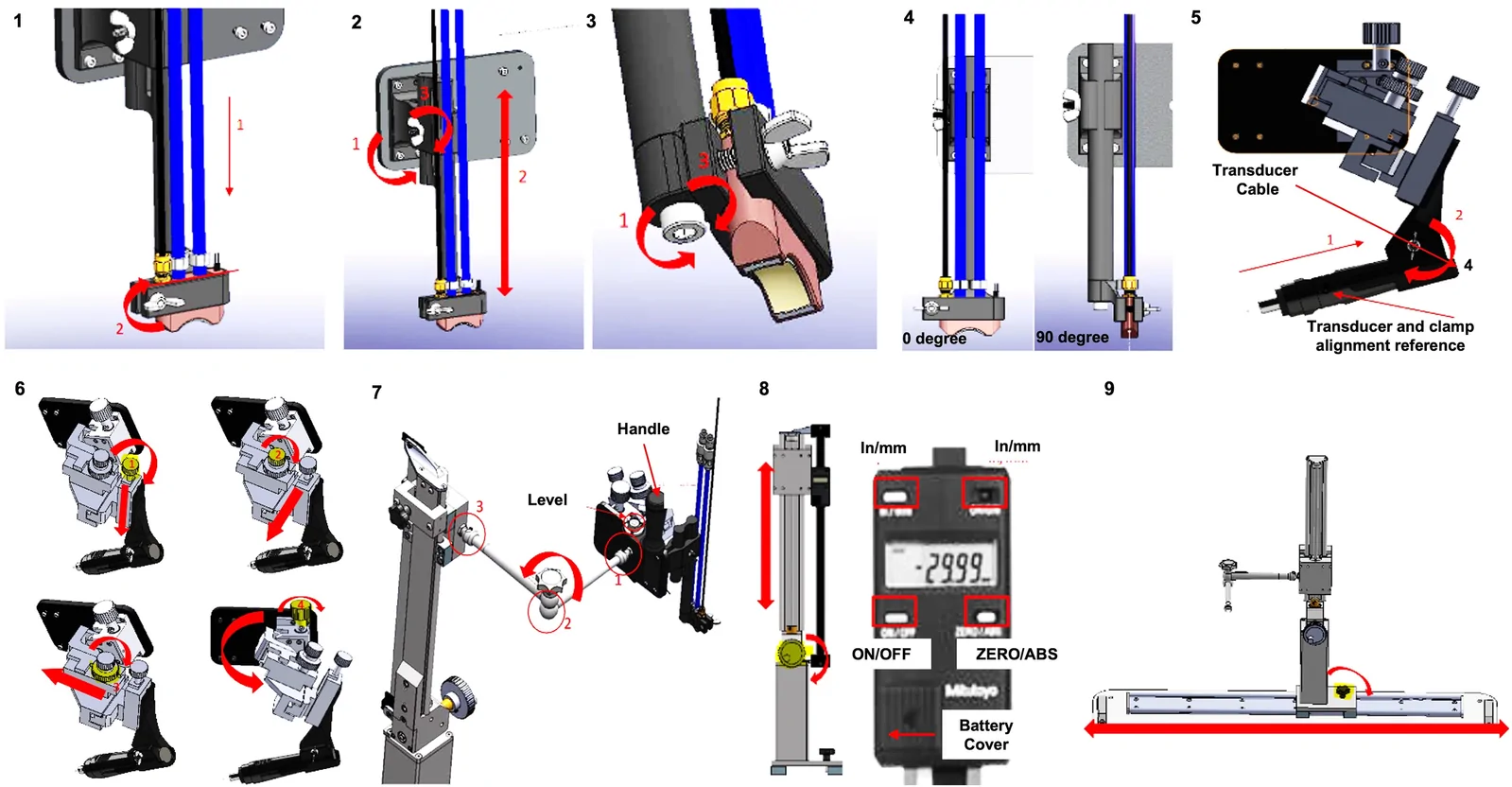
Transducer Set and Transducer Positioning Arm (1) Clamp for FUS transducer (Equipment Set Up, Step 1a). (2) z position adjustment for FUS transducer (Step 1b). (3) M5 socket head screw (Step 1c). (4) FUS transducer position on the clamp at 0 and 90 degrees angles (Step 1c). (5) dUS transducer clamp (Step 2a). (6) Adjustment of dUS transducer orientation (Step 2b). (7) Location of the joints of the articulated arm (Step 3). (8) Transducer set z-axis adjustment (Step 4). (9) Transducer set x-axis adjustment (Step 5).
  b. Adjust the z-axis position of the FUS transducer (Figure 3.2).
    i. Rotate the wing nut anti-clockwise to loosen the clamp and adjust the arm.
      **CRITICAL:** Hold on to the arm to prevent the transducer from dropping.
    ii. Adjust the z position of the transducer by moving the pole upward or downward.
    iii. Rotate the wing nut clockwise to tighten the clamp.
  c. Adjust the angle of the FUS transducer (Figures 3.3 and 3.4).
    i. Use a hex key to loosen the M5 screw (removal of the screw is not necessary).
      **CRITICAL:** Hold onto the arm to prevent the transducer from dropping.
    ii. Adjust the angle of the transducer about the z-axis, to either 0° or 90° to the right.
    iii. Use a hex key to tighten the M5 screw.
2. Assemble and position the dUS transducer.
  a. Attach the dUS transducer (Figure 3.5).
    i. Slide in the imaging transducer from the bottom.
    ii. Align the notch on the transducer to the pin on the clamp.
    iii. Rotate the wing nut clockwise to tighten the clamp until the transducer is fixed.
    iv. Plug in the transducer cable from the back, aligning the cable connector’s red dot to the front.
  b. Adjust the dUS transducer position (Figure 3.6).
    i. Rotate the first (bottom) knob to move along the z-axis: clockwise moves downward, anti-clockwise moves upward.
    ii. Rotate the second (top) knob to move along the y-axis: clockwise moves forward, anti-clockwise moves backward.
    iii. Rotate the third (bottom) knob to move along the x-axis: clockwise moves left, anti-clockwise moves right.
    iv. Rotate the fourth knob to rotate the transducer about the y-axis: clockwise rotates anti-clockwise, and anti-clockwise rotates clockwise.
3. Adjust the articulated (jointed-arm) dUS-FUS transducer positioning arm (Figure 3.7).
  a. Rotate the knob on the arm anti-clockwise to release the lock.
    **CRITICAL:** Release the lock carefully and hold on to the handle while releasing.
  b. Adjust the jointed arm position freely in the three joints.
  c. Rotate the knob clockwise to lock the position.
    ***Note:*** The arm releases each of the three joints progressively, starting with the joint closest to the transducer.
4. Adjust the transducer-set post along the z-axis (Figure 3.8).
  a. Rotate the reel to adjust the transducer set’s height (clockwise moves downward; anti-clockwise moves upward).
  b. Use the digital readout to monitor and set the post position.
    i. Press “ON/OFF” to turn the digital scale on or off.
    ii. Press “in/mm” to switch the display unit between inches and millimeters.
    iii. Press and hold “ORIGIN” for over one second to set the current location as the origin (for ABS mode).
    iv. Press or hold “ZERO/ABS” to switch between Incremental and Absolute modes.
      ***Note:*** In Incremental mode, an “INC” indicator appears in the top-left corner of the display and “ZERO/ABS” sets zero at any point; holding “ZERO/ABS” for more than two seconds switches to Absolute mode, in which the INC indicator disappears and measurements are taken from the origin.
    v. Slide left to remove the battery cover and replace the battery; the origin must be reset after the battery is replaced.
5. Adjust the transducer-set post along the x-axis (Figure 3.9).
  a. Rotate the knob anti-clockwise to release the lock.
  b. Move the post along the rail.
  c. Rotate the knob clockwise to lock.
6. Position the hydrophone stage.
  a. Move the stage along the x-axis (Figure 4.1).
    i. Rotate the knob anti-clockwise to release the lock.
    ii. Move the platform along the rail.
    iii. Rotate the knob clockwise to lock.

**Figure 4:**
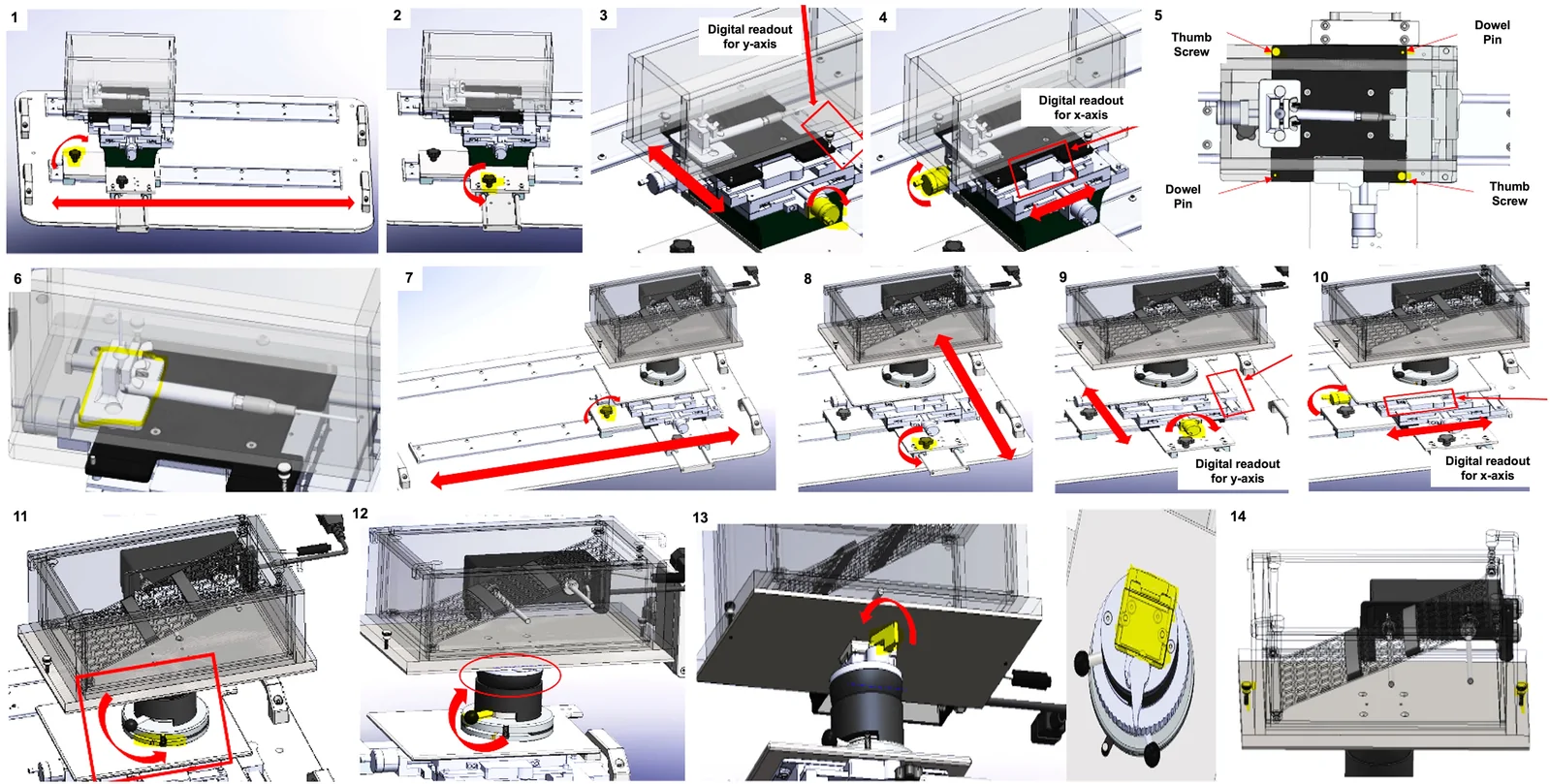
Hydrophone Tank Stage and Small Animal Tank Stage (1) Hydrophone platform, x-axis adjustment (Equipment Set Up, Step 6a). (2) Hydrophone platform, y-axis adjustment (Step 6b). (3) Hydrophone platform, y-axis fine adjustment (Step 6c). (4) Hydrophone platform, x-axis fine adjustment (Step 6d). (5) Hydrophone tank on the hydrophone platform (Step 6e). (6) A view of inside of hydrophone tank with hydrophone and hydrophone holder (Step 6f). (7) Small animal platform, x-axis adjustment (Step 7a). (8) Small animal platform, y-axis adjustment (Step 7b). (9) Small animal platform, y-axis fine adjustment (Step 7c). (10) Small animal platform, x-axis fine adjustment (Step 7d). (11) Magnetic lock of the small animal platform (Step 7e). (12) Ball joint and lock for angular adjustments (Step 7f). (13) Quick release lock (Step 7g). (14) Thumb screws for removal of small animal water tank (Step 7h).
  b. Move the stage along the y-axis (Figure 4.2).
    i. Rotate the knob anti-clockwise to release the lock.
    ii. Move the platform along the rail.
    iii. Rotate the knob clockwise to lock.
  c. Fine-tune the platform position in the y-axis (Figure 4.3).
    i. Rotate the front knob to fine-adjust the platform position in the y-direction (clockwise moves forward; anti-clockwise moves backward).
    ii. Refer to the digital readout on the right for the y-axis position (see Step 9 for digital scale settings).
  d. Fine-tune the platform position in the x-axis (Figure 4.4).
    i. Rotate the upper knob to fine-adjust the platform position in the x-direction (clockwise moves left; anti-clockwise moves right).
    ii. Refer to the digital readout on the front for the x-axis position (see Step 9 for digital scale settings).
  e. Remove and replace the hydrophone tank (Figure 4.5).
    i. Remove the two thumb screws.
    ii. Pick up and remove the tank.
    iii. Align the two pins and place the tank back.
    iv. Tighten the two thumb screws.
  f. Install the needle hydrophone in its holder (Figure 4.6).
    i. Pick up the hydrophone holder, which is designed for the Onda needle hydrophone (HNA-0400); two magnets under the holder guide it into the pocket.
    ii. Remove the two wing nuts and the clamping plate.
    iii. Remove or attach the hydrophone.
    iv. Place the clamping plate back and tighten the two wing nuts.
    v. Place the clamp in the slot in the tank.

**CRITICAL:** Do NOT touch the tip of the hydrophone.

7. Position the small-animal stage.
  a. Move the stage along the x-axis (Figure 4.7).
    i. Rotate the knob anti-clockwise to release the lock.
    ii. Move the platform along the rail.
    iii. Rotate the knob clockwise to lock.
  b. Move the stage along the y-axis (Figure 4.8).
    i. Rotate the knob anti-clockwise to release the lock.
    ii. Move the platform along the rail.
    iii. Rotate the knob clockwise to lock.
  c. Fine-tune the platform position in the y-axis (Figure 4.9).
    i. Rotate the upper knob to fine-adjust the platform position in the y-direction (clockwise moves forward; anti-clockwise moves backward).
    ii. Refer to the digital readout on the right for the y-axis position (see Step 9).
  d. Fine-tune the platform position in the x-axis (Figure 4.10).
    i. Rotate the upper knob to fine-adjust the platform position in the x-direction (clockwise moves left; anti-clockwise moves right).
    ii. Refer to the digital readout on the front for the x-axis position (see Step 9).
  e. Engage the magnetic lock (Figure 4.11).
    i. Turn the lever anti-clockwise to release the magnetic lock.
    ii. Move the tank freely on the platform.
    iii. Turn the lever clockwise to lock.
  f. Adjust the tank angle (Figure 4.12).
    i. Hold onto the water tank or remove the tank.
    ii. Switch the lever clockwise to release the lock SLOWLY.
      **CRITICAL:** The tank is heavy and full of water; hold the tank before releasing the lock to prevent water spills or the tank being dropped.
    iii. Adjust the water tank with the ball joint.
    iv. Switch the lever anti-clockwise to lock.
  g. Quick-release the water tank (Figure 4.13).
    i. Pull the lever under the tank holding plate to unlock.
    ii. Remove the tank with the holding plate.
    iii. Align the quick-release lock and push the tank down to lock.
      **CRITICAL:** Wires for the heating elements exit from the right rear of the tank. Disconnect the wire connectors before removing the tank.
  h. Remove the small-animal tank (Figure 4.14).
    i. Disconnect the heater plug from the back of the tank.
    ii. Remove the two thumb screws on the side of the water tank.
    iii. Remove only the water tank, without the metal tank holder.
    iv. Put the tank back in the tank holder and align the two screw holes on the side.
    v. Tighten the thumb screws.
  i. Control the water temperature (Figure 5.1).
    i. Locate the temperature control box next to the water tank.
    ii. Plug in the power cord.
    iii. Turn on the switch on the box.
    iv. Allow the temperature to rise to the desired value; refer to the digital display on the box for the desired and current water temperature.
    v. Turn off the switch on the box.
    vi. Connect or disconnect the temperature sensor and heater cords from the rear of the control box for ease of tank cleaning.

**Figure 5:**
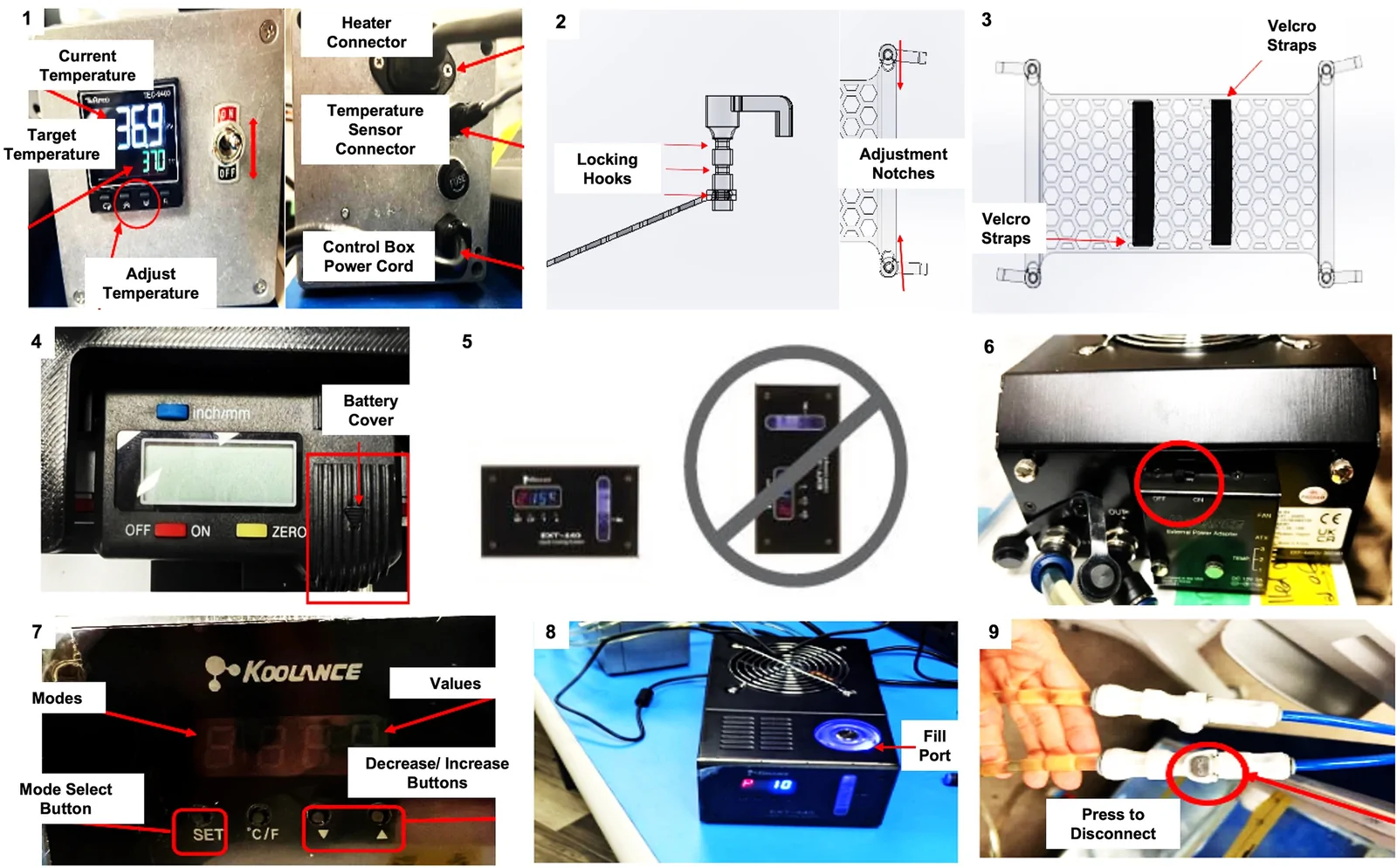
Water Temperature Control, Small Animal Bed, Digital Readouts for Fine Adjustments of stages, and Transducer Cooling System (1) Heater temperature control box (Equipment Set Up, Step 7i). (2) Adjustable animal bed showing height adjustment notches (left) and the locking mechanism for the adjustment hook (right) (Step 8a). (3) Animal bed with Velcro straps used to secure the animal to the bed (Step 8b). (4) XY stage digital readout (Step 9). (5) Orientation of cooling unit EXT-440 (Step 10a). (6) AC power adaptor for cooling unit (Step 10b). (7) LED display for the cooling unit (Step 10b). (8) Recommended setting for fan and flow is maximum (i.e., P 10, F10) with Coolant reservoir and filling port (Steps 10b–c). (9) Quick connection of hoses (Step 10d).
8. Adjust the small-animal bed.
  a. Adjust the inclination angle.
    i. Lift upward to remove the bed from the tank.
    ii. Slide the two higher hooks toward the center (Figure 5.2).
    iii. Adjust the hook position into one of the three slots (Figure 5.2).
    iv. Slide the hook toward the edge.
  b. Adjust the animal holding tie.
    i. Loosen or remove the tie (Figure 5.3).
    ii. Replace the tie with one of a different length (Figure 5.2).
9. Use the digital readout for the fine-adjust stages (Figure 5.4).
  a. Press “ON/OFF” to turn the readout on or off.
  b. Press “inch/mm” to switch the display unit between inches and millimeters.
  c. Press “ZERO” to set the current location as zero.
  d. Slide toward the arrow to remove the battery cover and replace the battery.
10. Set up the FUS transducer cooling system.
  a. Orient the cooling system correctly (Figure 5.5).
    **CRITICAL:**
    i. Keep the EXT-440 running upright to keep the reservoir air filtration functional.
    ii. Refer to the “EXT-440CU – Liquid Cooling System Manual – Koolance” for further information.
  b. Operate the cooling unit.
    i. Plug in the power cord.
    ii. Switch the cooling unit power ON/OFF (Figures 5.5 and 5.6).
    iii. Press “SET” to cycle through the modes listed in Table 1.

**Table 1:** Mode description of FUS transducer cooling system

Mode	Description
1	Temperature sensor #1 is displayed
2	Temperature sensor #2 is displayed
3	Temperature sensor #3 is displayed
4	All temperature sensors are cycled automatically
F	Fan settings are displayed. Automatic or 10 manual level (1-10).
P	The pump setting is displayed. 10 manual level (1-10)
7	All temperatures, fan, and pump settings are cycled automatically
    iv. Use the “increase setting” or “decrease setting” button to set the desired setting or mode; a “P 10” and “F 10” setting (maximum for both) is recommended (Figure 5.7).
  c. Fill the cooler reservoir with liquid coolant (Figure 5.8).
    i. Ensure all tubes and wires are connected.
    ii. Remove the large slot-headed screw with a screwdriver or large coin.
    iii. Place the cooling unit above all other liquid components to avoid spillage.
    iv. Pour liquid coolant LIQ-705CL-B slowly into the fill port; a non-conductive coolant MUST be used.
    v. Turn on the power to assist in the filling process.
    vi. Check the hose connections and tubes for folding, leakage, or blockage.
    vii. Replace the fill port on the reservoir.
      **CRITICAL:** Do not overtighten the fill port.
  d. Connect or disconnect the FUS transducer water lines (Figures 5.8 and 5.9).
    i. Turn off the cooling unit.
    ii. Press on the quick-connect joint and pull to disconnect.
    iii. Plug in to connect.
    iv. Turn on the cooling unit.
      ***Note:*** A small amount of liquid coolant may spill when disconnecting the hose. A shut-off valve on both sides of the connector prevents air from entering the system. The coolant is Koolance LIQ-705, a low-electrical-conductivity liquid coolant.
11. Set up co-registration of the focal depths of the dUS-FUS transducer.
  a. Set up the hydrophone.
    i. Assemble the hydrophone with a 90-degree SMA connector and pre-amplifier.
    ii. Place the assembly inside the hydrophone holder and hold it in place, avoiding contact with the hydrophone tip. This holder is designed to hold ONDA needle hydrophones (e.g., HNA, HNC types) (Figure 6.1).

**Figure 6:**
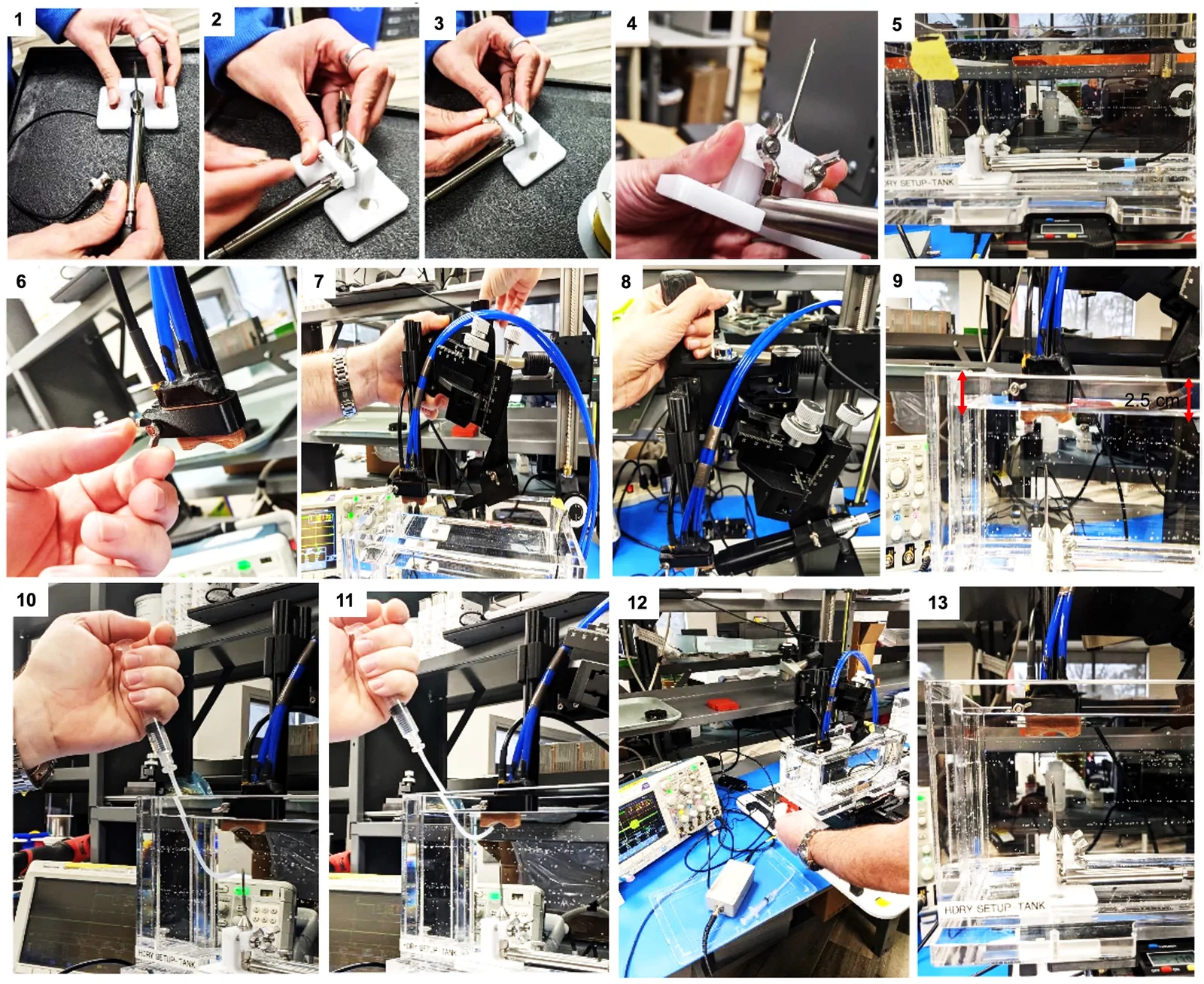
Setup for Co-registration of Focal Depths of dUS-FUS Transducer (1) Placement of hydrophone inside the clamp (Equipment Set Up, Step 11a.i–ii). (2 and 3) Securing needle hydrophone in the clamp (Step 11a.iii–iv). (4) Properly secured needle hydrophone in the clamp with magnetic base. (Step 11a.v). (5) Hydrophone placed in the Hydrophone water tank (Step 11a.vi). (6 and 7) Secure FUS transducer inside the clamp with Transducer assembly inside the clamp and on the hydrophone stage (Step 11b.i–ii). (8 and 9) Place transducer inside the water above the hydrophone by releasing the articulating arm. Make sure to hold onto the handle when releasing the arm. Simultaneously, the transducer is placed inside the water above the hydrophone. Recommended water level is 2.5 cm from the top of the tank (Step 11b.iii–iv). (10 and 11) Remove bubbles from the transducer face and hydrophone (Step 11b.v). (12 and 13) Adjust the x and y stages to align the transducer versus the hydrophone tip visually (Step 11b.vi).
    iii. Clamp the other side of the clamp in place while holding onto the assembly.
    iv. Add screws one at a time to the holder; do not tighten the screws until both sides are in place (Figures 6.2 and 6.3).
    v. Visually inspect to confirm the hydrophone is sitting square in the holder, with the tip not tilted (Figures 6.4 and 6.5).
    vi. Place the hydrophone in the designated slot in the water tank; two magnets at the bottom of the holder keep it in place. Avoid stressing the hydrophone’s strain relief (Figures 6.4 and 6.5).
    vii. Connect the hydrophone BNC cable to the DC power block.
    viii. Use another BNC cable to connect the output of the DC power block to the oscilloscope.
  b. Set up the FUS transducer.
    i. Place the FUS transducer inside the clamp, taking care with the water tubes and BNC cable so that they are secured properly and not under stress (Figures 6.6 and 6.7).
    ii. Confirm the dUS-FUS transducer assembly in the clamp matches Figure 5E.
    iii. Adjust the articulating arm to submerge the transducer into the water, holding on to the handle of the assembly when adjusting the arm (Figures 6.8 and 6.9).
    iv. Set the water level to the recommended 2.5 cm below the top of the tank (Figure 6.10).
    v. Inspect the hydrophone and transducer face for bubbles and remove any using a water syringe (Figure 6.11).
    vi. Perform a visual alignment and use the x- and y-direction stage knobs to place the center of the transducer above the hydrophone tip (Figures 6.12 and 6.13).
12. Set up the hydrophone measurement (Figure 7.1).
  a. Connect the signal generator output to the RF amplifier input.
  b. Connect the RF amplifier output to the impedance-matching circuit (RF IN) provided by the FUS transducer (frequency response of the matched transducer: Figure S1).
  c. Connect the ‘xdcr’ of the impedance-matching circuit to the transducer.
  d. Connect the ‘sync’ of the signal generator to the oscilloscope CH2 for triggering.
  e. Connect the hydrophone signal (output of the DC Block) to CH1 of the oscilloscope.
  f. Set the oscilloscope to trigger off the ‘sync’ signal.
  g. Set the signal generator for alignment (Figures 7.2, 7.3).
    i. Select burst mode with 20 cycles and a 1-ms burst period.
    ii. Set the amplitude to 27.5 mVpkpk.

**Figure 7:**
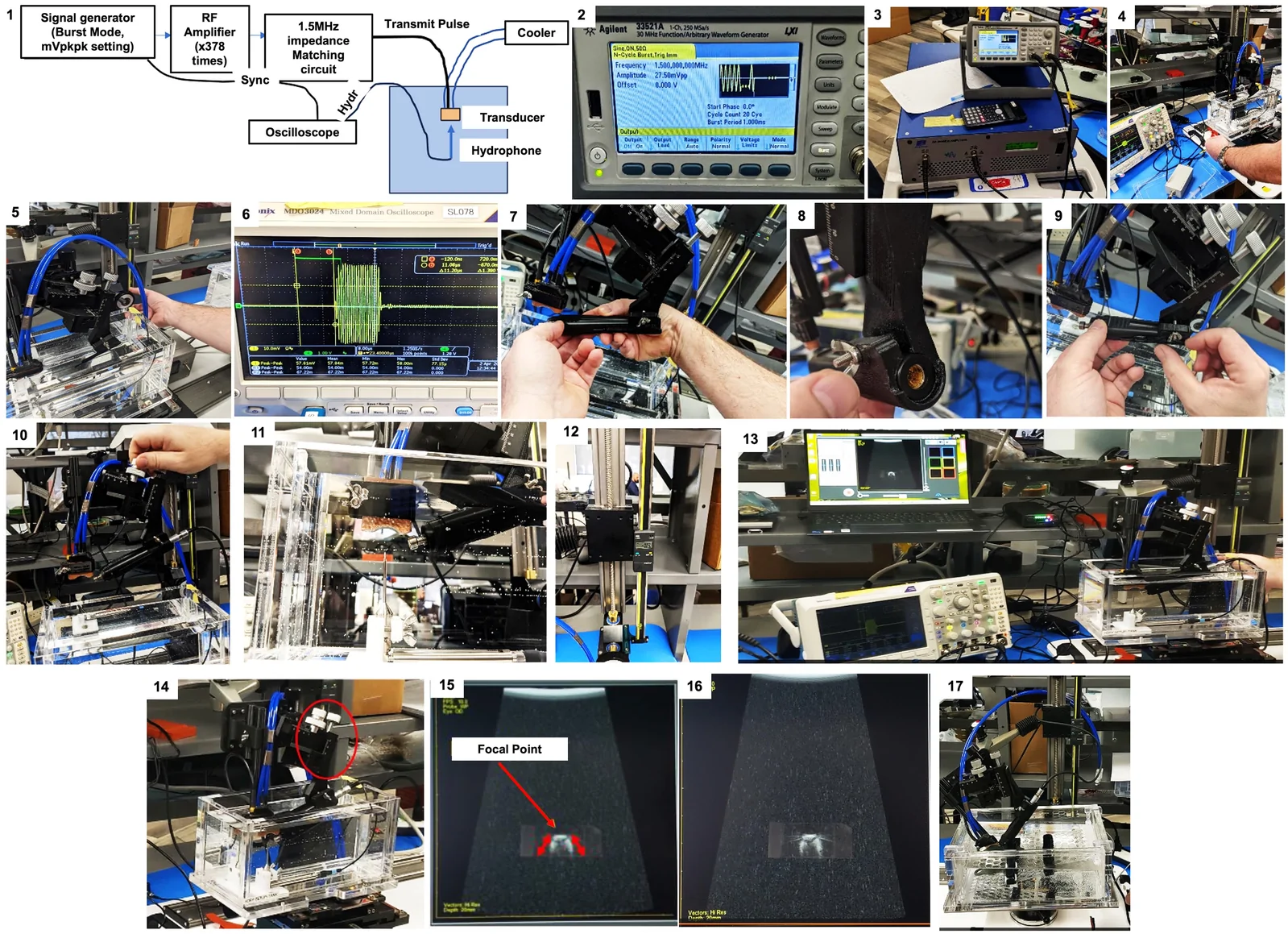
Hydrophone Measurements, Alignment of FUS Transducer versus Hydrophone and Co-registration of Focal Depths of dUS Versus FUS Transducer (1) Diagram for setup during hydrophone measurement (Equipment Set Up, Step 12a–f). (2 and 3) Setting of the signal generator for performing alignment step along with RF amplifier (Step 12g). (4) Adjusting x & y stages while monitoring the hydrophone signal on the oscilloscope to maximize (Step 13a–b). (5) Adjusting z stage to optimize the distance of transducer versus hydrophone while monitoring the hydrophone signal on the oscilloscope to maximize (Step 13c). (6) The hydrophone signal after the completion of alignment. Pay attention to the pkpk and TOF values (setting for the signal generator as shown in Figure 6B) (Step 13d). (7, 8, and 9) Placing the imaging transducer inside the clamp (Step 14c). (10, 11, and 12) Both transducers secured in the clamps of the arm. Where, submerge transducers inside water using the ‘z’; axis knob only until it reads zero (Step 14c–d). (13) Monitoring the image of the tip of the hydrophone while optimizing the angle and position of the imaging transducer (Step 14i). (14) Adjusting the angle and location of imaging transducer versus Focused transducer using shown knobs (Step 14i). (15 and 16) Needle Hydrophone tip after that the imaging transducer angle/position is optimized (Step 14j–k). (17) Slide over the arms to the imaging stage (Step 14l).

***Note:*** During alignment, the input voltage to the transducer can be as low as 10 Vpkpk; the RF amplifier (E&I 2100L) output to the transducer will be 27.5 mVpkpk × 378 = 10.39 Vpkpk.

***Note:*** Burst mode (20 cycles, 1-ms burst period; duty cycle ≈ 1.3%) is used only for alignment and hydrophone measurements. Continuous-wave operation (burst mode off) is used only for the *in vivo* exposure in Step 18e.

**CRITICAL:** For high-pressure measurements, connect the cooler tubes to the transducer water tubes and turn on the cooler. Set the signal generator to Burst mode with the voltage in mVpkpk, and check the output of the RF amplifier on the oscilloscope before connecting it to the transducer.

13. Align the FUS transducer with the needle hydrophone.
  a. Perform a visual alignment and place the center of the transducer on top of the tip, using the x- and y-axis knobs to move the hydrophone (Figure 7.4).
  b. Turn on the signal generator and monitor the hydrophone signal on the oscilloscope while aligning in the x- and y-directions (Figure 7.5).
  c. Determine the peak signal along the z-axis and verify the alignment using the time of flight (TOF).
    i. After determining the peak signal in the x- and y-directions, adjust the z-axis to also determine the peak signal.
    ii. Measure the TOF between the FUS transducer surface and the tip of the needle hydrophone.
    iii. Confirm that the TOF is approximately 11.1–11.2 μs, which is considered acceptable alignment.
      ***Note:*** Measuring the TOF reduces operator variability and increases accuracy. For this FUS transducer, the expected focal depth is ∼16.5 mm, so TOF = 16.5 mm / 1485 m/s = 11.1 μs when aligned.
  d. Confirm the alignment waveform (Figure 7.6).
    i. Set the signal generator to 27.5 mVpkpk (10.4 Vpkpk after amplification).
    ii. Read the peak-to-peak hydrophone voltage and the TOF on the oscilloscope.
      ***Note:*** The expected peak-to-peak hydrophone voltage for the HNA-0400 with pre-amp (AH-2020-100) at ‘0’ gain is 57.81 mVpkpk (57.8 mV / 0.1326 = 436 kPa), with a TOF of 11.2 μs.
  e. After alignment is complete, reset the three micrometers in the x-, y-, and z-directions to ‘zero’.
    **CRITICAL:** DO NOT move or adjust the articulating arm before the next step.
14. Co-register the focal depths of the dUS and FUS transducers.
  a. Turn off the signal generator.
  b. Use the z-axis knob (not the articulated arm) to raise the transducer out of the water.
  c. Place the dUS transducer in the clamp, adjusting its angle as needed; slide it up through the sleeve and tighten the screw, taking care not to hit the FUS transducer (Figures 7.7, 7.8, and 7.9).
    ***Note:*** The co-installed dUS and FUS transducers are shown in Figures 7.7–7.10.
  d. Lower the z-axis using the knob until the micrometer reads ‘zero’ (Figures 7.10, 7.11, 7.12, and 7.13).
  e. Inspect for air bubbles after submerging the dUS and FUS transducers (Figures 6G and 6H).
  f. Confirm that the x- and y-axis micrometers read ‘zero’.
  g. Turn on the signal generator and confirm that the hydrophone signal on the oscilloscope is peaked, as in Figure 7.6.
  h. Connect the imaging transducer cable to the laptop and start imaging.
  i. Adjust the imaging-arm knobs while monitoring the hydrophone-tip image (Figures 7.14, 7.15, and 7.16).
    **CRITICAL:** Be careful not to hit the FUS transducer, and do not submerge the end of the dUS transducer in the water.
  j. Confirm optimal positioning (Figures 7.14, 7.15, and 7.16).
    ***Note:*** The hydrophone-tip image should be maximized in intensity, with downward tails and a flat spot at the center.
    ***Note:*** At this point, the focal points of both transducers are co-registered; the hydrophone signal is maximized at a distance of ∼16.5 mm, and the hydrophone tip is imaged as in Figures 7.15 and 7.16.
    **CRITICAL:** Do not change the dUS transducer knobs after this step.
  k. Mark the imaging screen to annotate the location of the FUS transducer’s focus; the region of interest (ROI) for treatment must be placed at this location (Figures 7.15 and 7.16).
  l. Release the z-arm and slide it to the imaging side to perform the procedure.
    ***Note:*** The articulated arm may be adjusted as needed.
    **CRITICAL:** Do not change the position of the dUS transducer relative to the FUS transducer after co-registration is complete.
  m. Verify the cooling system and drive settings before each sonication.
    i. Confirm that the water tubes are connected to the cooling system, that the reservoir contains sufficient coolant, and that the cooler is turned on.
    ii. Turn the cooler on first and off last.
      **CRITICAL:** Keep the cooler on throughout the procedure to avoid damaging the FUS transducer from heat.
    iii. Confirm the input voltage to the FUS transducer (i.e., after the RF amplifier) before connection.
      ***Note:*** The maximum recommended driving voltage is 155 Vpkpk (410 mVpkpk on the signal generator with the E&I 2100L amplifier).
    iv. Confirm that the signal generator is set to the correct mode and parameters: burst mode (20 cycles, 1-ms burst period) for alignment and hydrophone measurements, or continuous-wave mode (burst off) only for the *in vivo* exposure in Step 18e.
      ***Note:*** Continuous-wave operation is not recommended for alignment, hydrophone measurements, or any exposure other than the *in vivo* exposure described in Step 18e.
  n. After the procedure, confirm that the co-registration of the dUS and FUS transducers is unchanged.
    i. Slide the post back onto the hydrophone tank.
    ii. Reset the signal generator to the alignment settings (burst mode, 20 cycles, 1-ms burst period, 27.5 mVpkpk).
    iii. Check the hydrophone signal on the oscilloscope and confirm that it matches Figure 7.6.

### dUS-FUS system setup: Integration of vacuum tank, dUS-FUS transducer assembly, hydrophone system, and temperature-controlled small-animal tank

**Timing: ∼2 h 30 min**

This procedure describes the complete setup of the dUS-FUS system before transducer calibration and *in vivo* experiments. It includes establishing the required system connections, preparing degassed deionized water, setting up the hydrophone tank and FUS transducer cooling system, and preparing the temperature-controlled small-animal tank for subsequent image-guided FUS procedures.

15. Wire and connect the dUS-FUS components, as shown in Figures 8.1 and 8.2.

**Figure 8:**
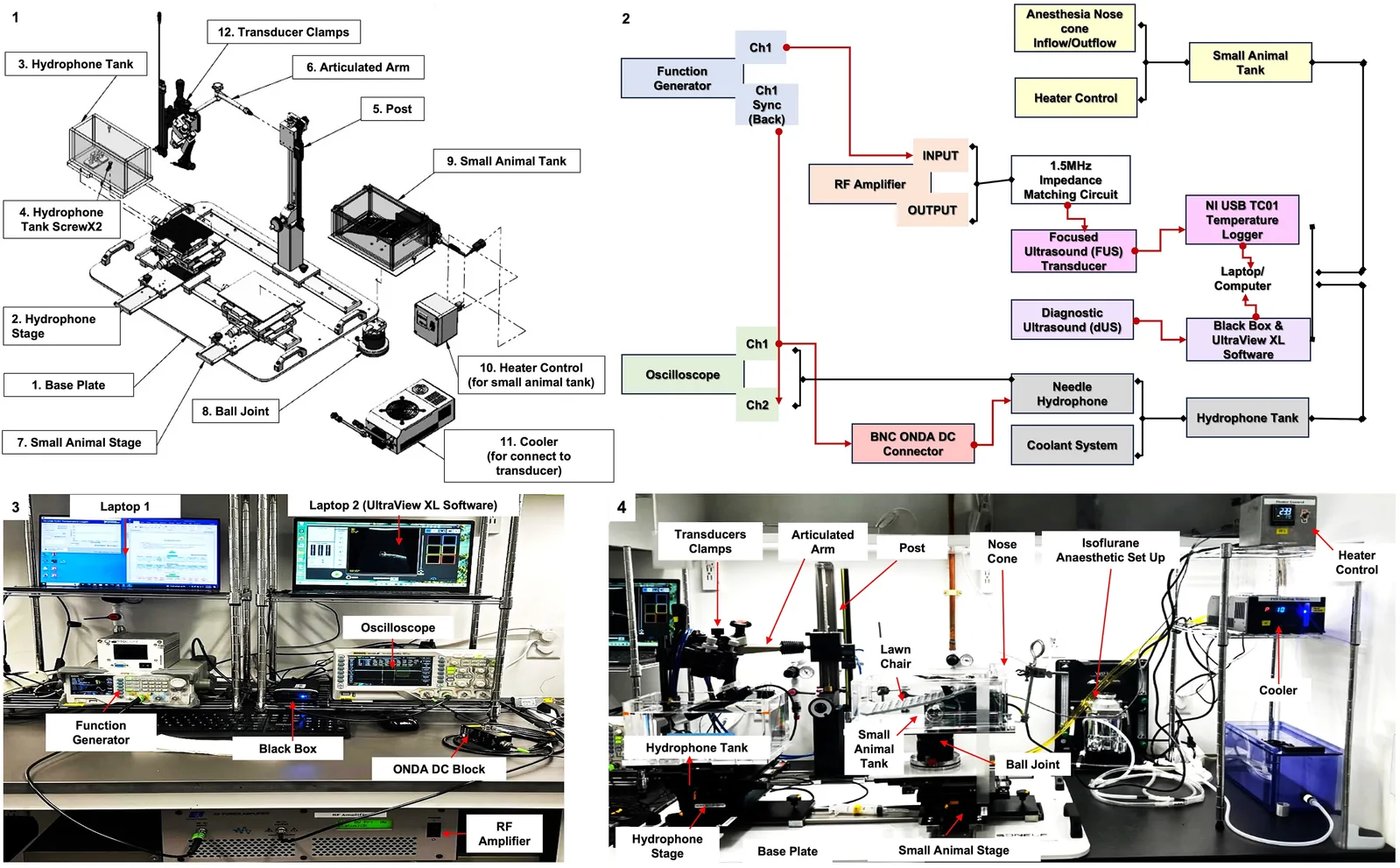
dUS-FUS System Set-Up (1) Pictorial depiction of the Small Animal System Assembly (Step 15), (2) Detailed schematic illustrating the wiring and connections of the dUS-FUS components (Step 15), (3, 4) The original layout of the fully assembled dUS-FUS system shows the small animal system assembly with connections and wiring (Step 15)
16. Prepare the vacuum, hydrophone, and small-animal tanks.
  ***Note:*** This step includes water degassing, transducer cooling, hydrophone positioning, and establishment of a 37°C water environment for animal experiments (∼30 min).
  a. Fill the vacuum tank (Figure 9.1).
    i. Turn off the vacuum and release the pressure by opening the pressure valve. Unfasten all hooks (front, back, right, and left) and open the vacuum tank lid.
    ii. Using a funnel pitcher, fill both the hydrophone tank and the small-animal tank with de-ionized water.
    iii. Refill the vacuum tank with replacement de-ionized water to ensure degassed water is available for future experiments.
    iv. Close the vacuum tank lid securely, ensuring the pressure valve and all hooks (front, back, right, and left) are properly tightened, then restart the vacuum once the lid is secured.

**Figure 9:**
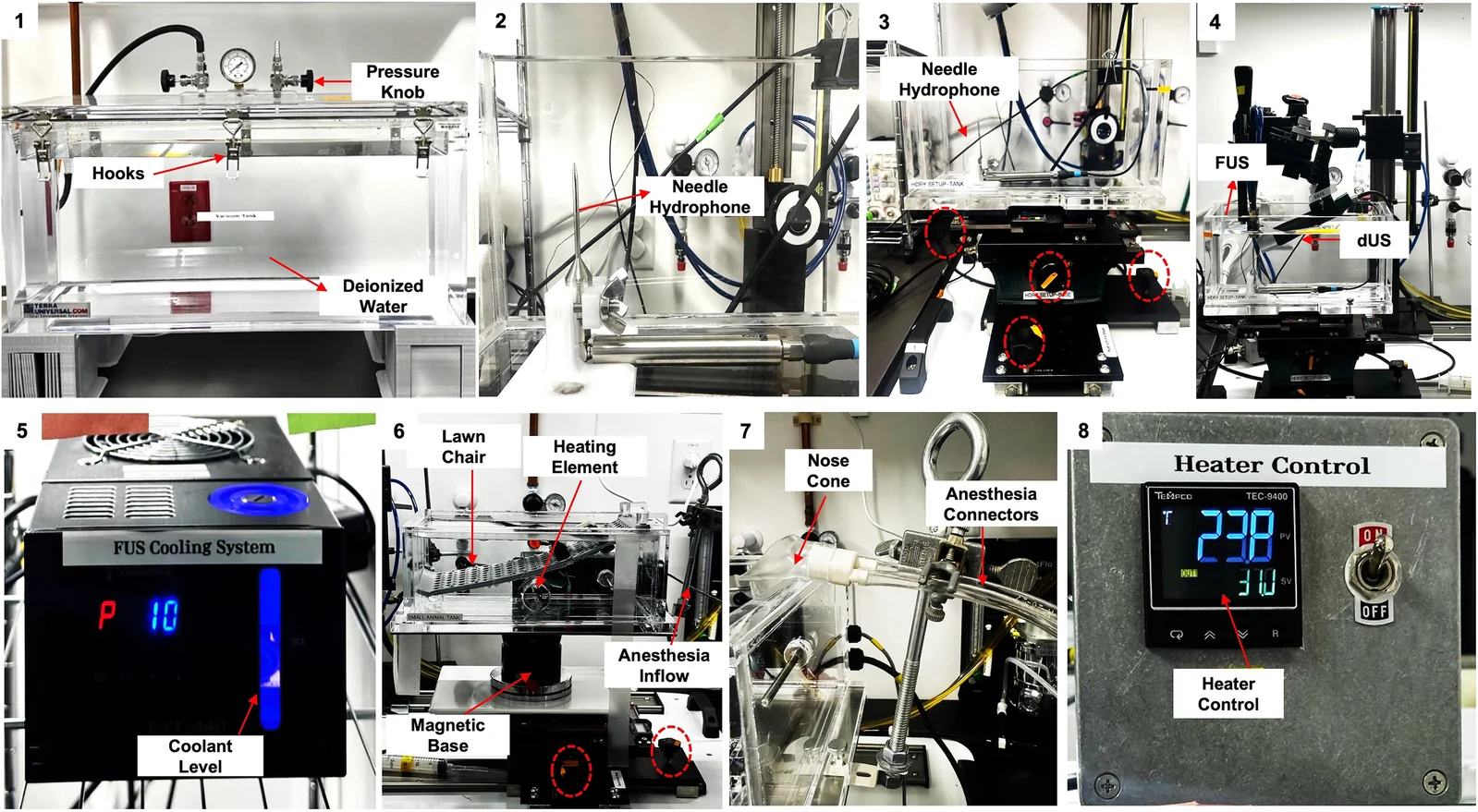
dUS-FUS System Set-Up: Integration of Vacuum Tank, Transducer Assembly, Hydrophone System, and Temperature-Controlled Small Animal Tank (1) Vacuum tank for filling the hydrophone and small animal tanks with deionized water, featuring hooks, knobs, and lids (Step 16a). (2) Needle hydrophone positioned inside the hydrophone tank (Step 16b.i). (3, 4, and 5) Hydrophone tank with adjustment knobs for precise alignment. Along with the dUS-FUS transducer set placed above the needle hydrophone, alongside the FUS cooling system filled with coolant to a level midway between the “Min” line and the top of the window, with the flow set to “10.” (Step 16b.ii–iv). (6, 7, and 8) Small animal tank equipped with a nose cone for anesthesia delivery and an integrated heating control system (Step 16c.i–iv).
  b. Fill the hydrophone tank and set up the FUS transducer cooling system.
    i. Before filling the tank, ensure the needle hydrophone is securely clamped in its holder, with the holder positioned in the indentation and held in place by the embedded magnets (Figure 9.2).
    ii. Fill the hydrophone tank with degassed, de-ionized water up to the black mark on the left and right sides (approximately 2 cm from the top) (Figures 9.2 and 9.3).
    iii. Turn on the FUS transducer cooling system, ensuring there is enough coolant (level approximately halfway between the “Min” line and the top of the window) and that the flow is set to “10” (Figure 9.4).
    iv. After filling the hydrophone tank and turning on the coolant, carefully lower the FUS-dUS transducers into the tank (Figure 9.5).
      **CRITICAL:** Avoid touching or pouring water directly onto the tip of the needle hydrophone; pour water slowly along the sides of the hydrophone tank. Use a syringe to remove any air bubbles in the hydrophone tank and from the FUS transducer face, avoiding contact between the syringe and the curved face of the FUS and diagnostic transducers.
  c. Fill the small-animal tank and set the temperature control box.
    i. Fill the small-animal tank with de-ionized water from the vacuum tank, ensuring sufficient space for the rat along with a nose cone for maintenance of anesthesia (Figure 9.6 and 9.7).
    ii. To prevent burns to the animal, position a small (25 or 50 mL) beaker beneath the lawn chair around the heating element so the heating element does not directly contact the lawn chair (Figure 9.6).
    iii. Keep a small container of degassed, de-ionized water nearby for quick use in case of spills from the animal tank.
    iv. After filling the tank, activate the heater and allow it to reach 37°C; bubbles will form in the heated water (Figure 9.8).
      **CRITICAL:** Pour water slowly from the sides of the small-animal tank and remove any air bubbles using a syringe. Do not turn on the heater until the tank is filled with water and turn on the heater control for the small-animal tank 1 to 1.5 h before the experiment.

### Setup for co-registration of focal depths of the FUS transducer and imaging transducer

**Timing: ∼30 min**

This procedure describes co-registration of the FUS and dUS transducers using a needle hydrophone to align their focal depths and verify alignment based on hydrophone signal and time-of-flight measurements.

17. Verify all cable connections and power on the function generator (FG), oscilloscope, RF amplifier, and hydrophone before proceeding (Figure 10).
  a. Wire the function generator (FG).
    ***Note:*** The FG converts electrical signals into ultrasound waves, producing specific waveforms or pulsed signals to control transducer characteristics such as pressure, intensity, cycles, and pulse duration.
    i. Connect ‘Ch1’ to the ‘INPUT’ of the RF amplifier and connect ‘Ch1 Sync’ (located at the back) to ‘Ch2’ on the oscilloscope.
    ii. Power on the FG and press “Store”, then press the blue “Browse” button, select the pre-set configuration ‘FUS1.RSF’, and press “Read”. This configuration (burst mode, 20 cycles, 1 ms, 27.5 mV peak-to-peak, 50 Ohm) is used for testing the FUS and aligning the transducers.
    iii. Switch off ‘Burst’ mode to verify the mV and MHz settings, then re-enable ‘Burst’ mode for alignment (Figure 10.1).
  b. Wire the oscilloscope.
    ***Note:*** The oscilloscope measures acoustic parameters, visualizes waveforms, ensures proper alignment, and verifies signal quality from the transducer, displaying voltage, amplitude, intensity, pulse duration, and pressure in real time.
    i. Connect ‘Ch1’ to the BNC connector on the back of the Onda DC Block, ensuring a 50-ohm terminator is attached to ‘Ch1’ and the Onda BNC cable is properly connected.
    ii. Connect ‘Ch2’ to ‘Ch1 Sync’ on the back of the FG.
    iii. Switch on the oscilloscope and confirm the scale in the lower-left corner reads 2 = 5.00 V (blue), with the upper-right corner displaying ‘H 5.00 μs’ and a scale of 20.0 mV (Figure 10.2).
  c. Wire the needle hydrophone.
    ***Note:*** The needle hydrophone converts pressure fluctuations into electrical signals, which are analyzed to determine pressure amplitude, intensity, beam patterns, total power, and other field characteristics, and is used to align the dUS-FUS transducer setup.
    i. Ensure the hydrophone is always submerged in water when powered ON (Figure 10.4).
    ii. Secure the needle hydrophone to the 90-degree connector, and attach the other end of the connector to the hydrophone preamp (Figure 10.4).
    iii. Connect the cable from the needle hydrophone to the hydrophone preamp, then link the preamp to the ‘Hydrophone Preamp’ port on the bottom of the Onda DC Block.
    iv. Attach one end of the second cable to the back of the DC Block and the other end to a BNC connector.
    v. Use ‘low gain’ for the Onda DC block and connect the power cord to the ‘DC In’ port on the Onda DC Block.
  d. Set up diagnostic ultrasound (dUS), the black box, and UltraViewXL software.
    ***Note:*** dUS is a non-invasive technique for anatomical and functional imaging that identifies target tissues for FUS by estimating the focal zone in the region of interest. Using UltraView XL software, dUS enables real-time imaging of the targeted site, such as the sciatic nerve discussed in this protocol.
    i. Turn on the laptop and log in. Power on the black rectangular box connected to the dUS before launching the UltraViewXL software on the dUS laptop (Figure 10.5).
    ii. Ensure the dUS transducer head is properly aligned with the needle hydrophone (Figures 10.4 and 10.5).
    iii. Check the alignment from the side to ensure the dUS transducer is centered on the hydrophone tip (Figures 10.4 and 10.5).
    iv. Position the tip of the hydrophone over the midpoint mark on the UltraViewXL software screen: approximately 12 mm (vertical bar) and 3 mm (horizontal bar) (Figure 10.5).
  e. Wire the RF amplifier.
    i. Before turning on the RF amplifier, ensure the BNC cables are securely plugged into both the RF input and RF output connections.
    ii. Power on the RF amplifier for the dUS-FUS transducer alignment (Figure 10.3).
  f. Align the dUS and FUS transducers using the oscilloscope signal (Figures 10.1–10.5).
    i. Press the ‘Output1’ button on the FG to activate the FUS, ensuring the correct parameters are set on the FG, including 50 Ohm.
    ii. Use the oscilloscope to locate the center of the Focal Zone (FZ).
    iii. Adjust the x-, y-, and z-axes individually to maximize the hydrophone signal on the oscilloscope.
    iv. Allow the signal to stabilize.
      ***Note:*** The initial reading may reach approximately 91.2 mVpp or higher (for our custom-built FUS transducer) immediately after the RF amplifier is turned on.
    v. Confirm that the stabilized hydrophone signal is within 88.0–89.6 mVpkpk (voltage criterion).
    vi. Measure the TOF from the FUS transducer surface to the tip of the needle hydrophone (which now corresponds to the middle of the focal zone) by pressing “Cursor” on the oscilloscope.
    vii. Position the left cursor at the beginning of the blue trigger-signal spike.
    viii. Position the right cursor where the yellow hydrophone signal begins to deviate from baseline.
    ix. Read the “BX” value, which is the TOF, and confirm that it is within 11.1–11.2 μs.
      ***Note:*** A TOF of 11.1–11.2 μs corresponds to a focal distance of ∼16.5 mm (11.1–11.2 μs × 1485 m/s ≈ 16.5 mm).
      **CRITICAL:** Alignment is complete only when both the voltage and TOF criteria are satisfied.
    x. After obtaining the image (Figure 10.5), double-check the alignment of the dUS with the tip of the hydrophone using a straightedge ruler along the side of the tank.
    xi. Confirm that the tip of the hydrophone (and thus the FUS focal zone) is approximately 12 mm from the dUS transducer, matching the focal depth of the 50 MHz dUS transducer (Figures 10.4 and 10.5).

**Figure 10:**
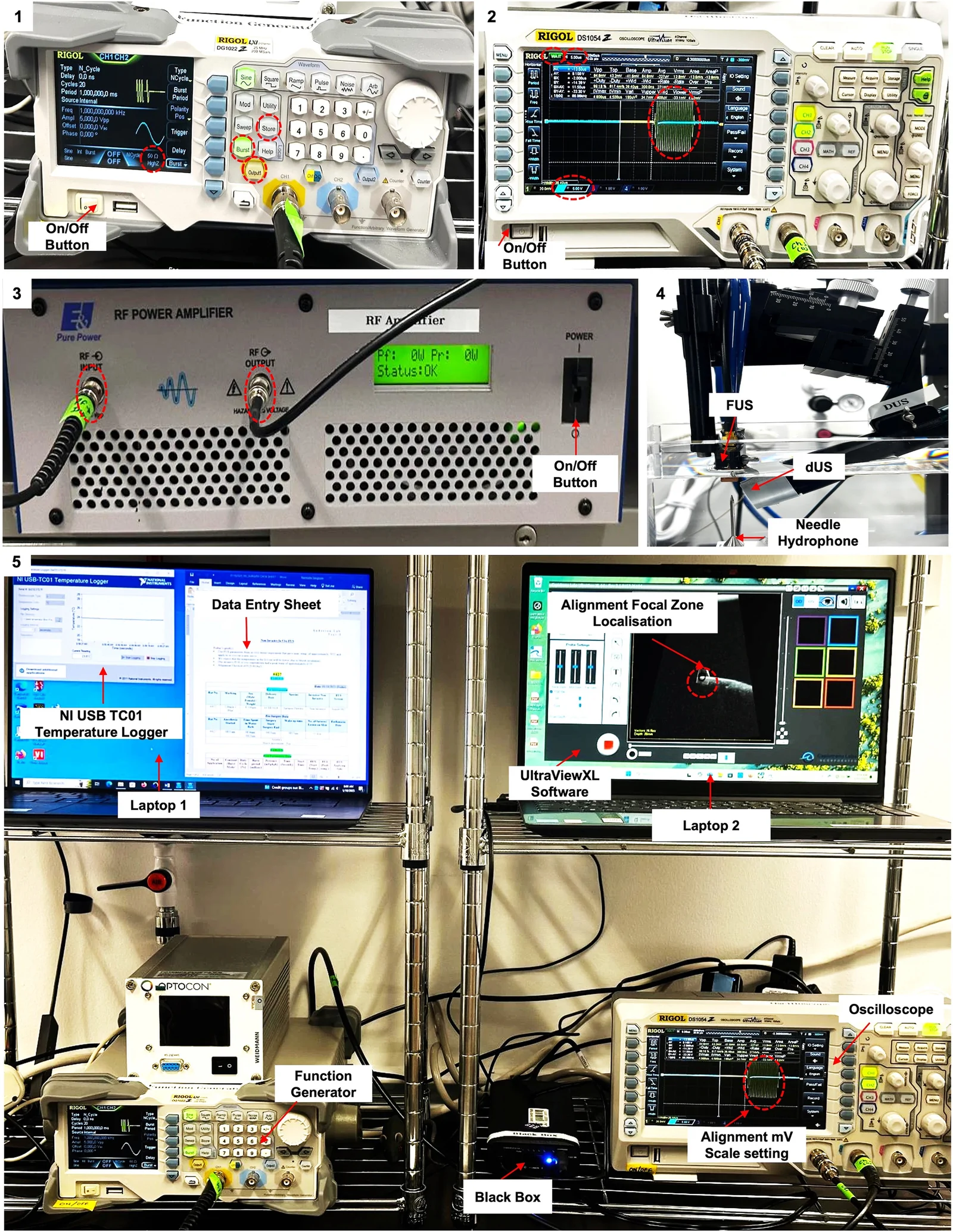
Setup for Co-registration of Focal Depths of dUS-FUS Transducer (1) Function Generator connections and configuration for co-alignment (Step 17a). (2) Oscilloscope wiring and scale settings for co-alignment (Step 17b). (3) RF Amplifier activation and connections (Step 17e). (4) Needle hydrophone and preamp connections with dUS-FUS transducer alignment (Steps 17c–d). (5) Laptop system configured for Time of Flight (TOF) measurement, temperature monitoring, and data logging. Black box integrated with UltraView XL software for real-time imaging and datasheet documentation (Steps 17d and 17f).

### *In vivo* non-invasive setup, dUS imaging, and FUS application

**Timing: ∼1 h**

This procedure describes the *in vivo* dUS-guided FUS workflow, including animal preparation and positioning, target tissue localization, FUS transducer temperature monitoring, FUS application, and post-procedure system cleanup. Once the wiring and alignment of the dUS-FUS transducers are complete, use the setup on the rat for imaging and FUS application, culminating in a hindpaw incision to establish an acute pain model (Figure 11.1).

18. Perform the *in vivo* dUS-guided FUS procedure.
  a. Anesthetize the animal and remove hair from the target site.
    i. Weigh the rat.
    ii. Induce general anesthesia in the induction chamber with 3% isoflurane delivered in 100% oxygen at a flow rate of 2 L/min.
    iii. Maintain anesthesia with 2% isoflurane in 100% oxygen at 1–1.5 L/min through the nose cone, adjusting the flow rate for the rat’s body weight (200–250 g).
    iv. Shave the leg near the right/left femur and biceps femoris using a trimmer to expose the skin (Figure 11.2).
    v. Apply Veet Sensitive Formula hair-removal cream for 10–15 min, then thoroughly clean the area using wet and dry wipes (Figure 11.2).
      **CRITICAL:** Ensure the rat remains anesthetized under the nose cone with isoflurane active throughout the procedure.
  b. Place the animal in the small-animal tank and switch to tank anesthesia.
    i. After removing the fur, switch off the induction-chamber anesthesia and activate the small-animal tank anesthesia system, maintaining isoflurane at 2% in 100% oxygen at 1–1.5 L/min.
    ii. Place the rat in the small-animal tank on the lawn chair, securing it in a right/left lateral position, with the snout on the isoflurane-maintained nose cone and the body submerged in degassed water at 37°C (Figure 11.3).
      **CRITICAL:** Use a circular ring to stretch and secure the leg (region of interest near the sciatic nerve) tightly, ensuring uniform surface tension, adequate blood flow, and minimal movement during FUS application. Adjust the lawn chair and rat position for optimal fit, adding a solid platform beneath the chair if necessary.
  c. Transfer the dUS-FUS transducers to the small-animal tank.
    i. After properly positioning the animal, use the transducer’s UP knob to lift the aligned dUS-FUS transducers from the hydrophone tank (Figure 11.4). Slide them to the small-animal tank and lower them using the DOWN knob, positioning them perpendicularly over the rat’s leg to image the sciatic nerve (Figure 11.5).
      **CRITICAL:** Slide the dUS-FUS transducers gently from the hydrophone tank to the small-animal tank to prevent bumps or misalignment, and ensure the transducers are fully submerged in water without touching the animal's skin.
  d. Log the FUS transducer temperature (Figure 11.7).
    i. Turn on the NI USB-TC01 temperature logger to monitor the temperature of the FUS transducer during FUS application (Figure 11.7).
    ii. Navigate to the designated directory to save the temperature data, select the CH1 channel, and click “Start Logging Data” to continuously record the temperature throughout the experiment (Figure 11.7).
    iii. Stop the sonication immediately (turn off the function generator output) if the logged transducer temperature exceeds 40 °C.
      ***Note:*** Continuously monitor the process to verify proper temperature recording in the designated folder and remember to stop logging on the NI USB TC01 temperature logger once the FUS application/experiment is complete.
      ***Note:*** The thermocouple measures the temperature of the face of the FUS transducer, not the temperature of the tissue. Tissue temperature is not measured on this platform (see Limitations).
  e. Locate the tissue of interest using dUS and administer FUS.
    i. Position the dUS transducer over the desired anatomical region and press start on the UltraViewXL software to begin real-time scanning. Continuously monitor the screen to identify the tissue of interest; in our proof-of-concept study, we targeted the seed-shaped sciatic nerve near the biceps femoris of the rat’s hind limb (Figure 11.6).
    ii. Adjust the dUS transducer by gently moving the small-animal tank (unscrewing it from the magnetic base) to confirm the sciatic nerve’s position beneath or near the biceps femoris. Once located, stabilize the dUS-FUS transducer at that position (Figure 11.5).
    iii. Adjust the base of the small-animal tank via the magnetic locks to obtain a back-and-forth view of the nerve in the UltraView XL image and confirm that the identified structure is the nerve.
    iv. Confirm that the dUS-FUS transducers are fully submerged in water and remove any air bubbles from the FUS transducer lens before starting.
    v. Load the *in vivo* preset ‘fusrat1.RSF’ on the function generator: press “Store”, press the blue “Browse” button, select the file, and press “Read”.
    vi. Verify that the function generator is set to 50 Ω, 1.5 MHz, and 250 mVpkpk, with burst mode off (continuous wave, 100% duty cycle), and set the exposure duration to 60 s (Figure 10.1).
      ***Note:****In vivo* acoustic exposure: a 1.5-MHz continuous sinusoidal wave (100% duty cycle; burst mode off) applied for 60 s. The generator setting of 250 mVpkpk (≈94.5 Vpkpk at the transducer after the 378× RF amplifier gain) corresponds to a calibrated focal pressure of 6.65 MPa, measured in degassed water at room temperature in the free field at the focus (∼16.5 mm) with the HNA-0400 needle hydrophone and pre-amp (sensitivity 0.1326 mV/kPa) during the pressure–voltage calibration (Figure S3).
      ***Note:*** The spatial-peak pulse-average intensity (ISPPA) was approximately 1436 W cm^−2^, calculated from the sinusoidal peak acoustic pressure amplitude of 6.65 MPa using I = p^2^/(2ρc), with ρc = 1.54 × 10^6^ kg m^−2^ s^−1^ under the plane-**wave** approximation. Because the exposure was continuous wave (100% duty cycle), the spatial-peak temporal-average intensity (ISPTA), averaged over the sonication interval, equaled ISPPA.
      ***Note:*** Continuous-wave operation corresponds to a 100% duty cycle. In burst mode (used only for alignment), the number of cycles per burst = frequency (cycles/second) × burst period (seconds) × duty cycle (fraction); for example, 1.5 MHz × 1 ms × 0.0133 ≈ 20 cycles.
      **CRITICAL:** Before applying FUS to the animal, double-check all parameters, confirm that the cooler is on, and confirm that the dUS-FUS transducers are fully submerged in water during application.
    vii. Press the output button on the FG and start the timer (Figures 10.1–10.5).
    viii. Administer FUS to the animal for 60 s.
      **CRITICAL:** Avoid contact between the transducers and the animal’s skin during the procedure to prevent FUS absorption in the skin and reduce the risk of burns or lesions from the mechanical effects of FUS at high pressure or intensity.
      **CRITICAL:** If skin blanching, erythema, blistering, or a lesion is observed, or if the logged transducer temperature exceeds the safety cutoff, stop the sonication immediately by turning off the FG output and follow the stop-and-check procedure in Troubleshooting (Problem 8). Do not move the transducers and the animal closer together and do not change the acoustic parameters ad hoc.
    ix. Once the exposure is complete, turn off the FG output and the temperature logger.
    x. Raise the dUS-FUS transducers away from the rat’s leg with the UP knob.
    xi. Turn off the anesthesia and transfer the animal to the surgery setup for the hindpaw incision surgery under anesthesia (Step 19).
  f. Follow the post-FUS administration protocol.
    i. After FUS application, slide the dUS-FUS transducers back into the hydrophone tank for realignment as described above.
    ii. Power off the following equipment: dUS black box, dUS laptop, small-animal tank heater, oscilloscope, FG, RF amplifier, FUS cooling system, and NI USB TC01 temperature logger.
    iii. Clean the small-animal water tank, lawn chair, and hydrophone tank.
      **CRITICAL:** Be cautious when cleaning the hydrophone tank and carefully remove the needle hydrophone without causing damage. Use a squeeze bottle with MilliQ water to gently rinse the dUS-FUS transducers, then dry the tanks and equipment carefully using wipes or tissues.

**Figure 11:**
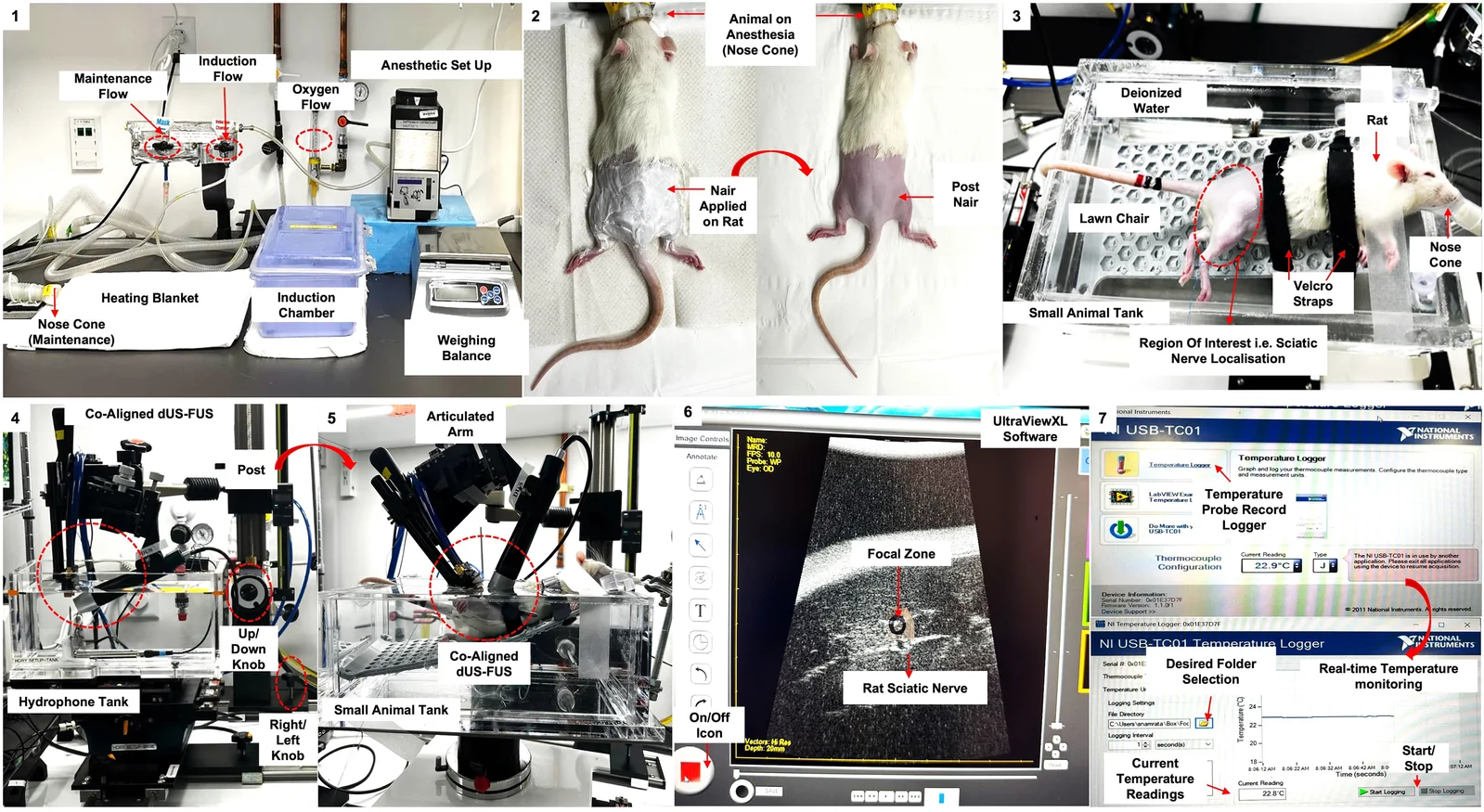
*In vivo* Non-Invasive Set-Up, dUS Imaging, and FUS application (1) Full experimental setup for animal preparation for FUS application (Step 18). (2) Anesthesia administration and hair removal at the target site (Step 18a). (3) Placement of the animal in the small animal tank with continuous anesthesia (Step 18b). (4 and 5) Transfer of dUS-FUS transducers from hydrophone tank to the small animal tank (Step 18c). (6) Localization of the sciatic nerve using dUS for FUS administration (Step 18e). (7) Real-time temperature monitoring and data logging with the FUS transducer temperature probe (Step 18d).

### *In vivo* hindpaw incision model for acute pain and behavioral assessment

**Timing: ∼3 h (for step 19)**

**Timing: ∼30 min per animal, including 15 min of acclimation followed by five trials with a maximum cutoff of 20 s per trial and a 3-min interval between consecutive trials (for step 20a)**

**Timing: ∼10 min per animal, including 5 min of handling followed by three trials with a 1-min interval between consecutive trials (for step 20b)**

**Timing: ∼1–2 min per animal (for step 20c)**

**Timing: ∼1–2 min per animal (for step 20d)**

This procedure describes establishment of the hindpaw incision model following FUS application, including surgical preparation, incision and wound closure, post-operative recovery, and subsequent behavioral assessment.

19. Establish the acute pain model.
  Following the transcutaneous application of FUS to the sciatic nerve, remove the rat from the small-animal tank and create an acute incisional injury using the hindpaw incision model for acute pain, as outlined by Brennan et al.^38^ Maintain normothermia throughout the procedure using a feedback-controlled heating system with continuous closed-loop temperature regulation.
  a. Ensure the surgical area and instruments are properly prepared and sterilized.
    ***Note:*** All experimental protocols involving live animals must receive approval from the Institutional Animal Care and Use Committee (IACUC).
  b. Anesthetize the rat using an isoflurane anesthetic system via a nose cone and place it on a heating pad maintained at 37°C to prevent hypothermia.
    **CRITICAL:** Confirm the depth of anesthesia by checking for the absence of a paw withdrawal reflex in response to a toe pinch.
  c. Apply a small amount of eye lubricant to both eyes to prevent dryness during the procedure.
  d. Clean the hindpaw twice using an alcohol swab and apply povidone-iodine with a cotton-tipped applicator.
  e. Using a sterile scalpel, create a 1 cm longitudinal incision through the skin and fascia, beginning near the heel and extending toward the toes.
  f. Carefully lift and make a longitudinal cut in the plantaris muscle, ensuring its origin and insertion points remain intact.
    **CRITICAL:** Take care to avoid injuring major blood vessels or nerves to minimize excessive bleeding or irreversible nerve damage. Apply gentle pressure or use sterile gauze to control any minor bleeding.
  g. Ensure hemostasis, then close the wound with non-absorbable sutures in a discontinuous pattern.
  h. Ensure the wound is securely closed to prevent infection and promote healing.
    **CRITICAL:** Apply antibiotics to the incised area to avoid infection.
  i. Allow the animal to recover from anesthesia in a warm, quiet environment.
    **CRITICAL:** Monitor the animal for signs of distress, infection, or complications.
20. Conduct behavioral assessment of sensory and motor function.
  ***Note:*** Neurobehavioral assessments evaluate the sensory and motor effects of FUS, focusing on its potential to achieve non-invasive, reversible peripheral nerve blockade (PNB) with preferential inhibition of nociceptive fibers. Four tests are used: (a) the modified Hargreaves test for C-fiber-mediated thermal hyperalgesia; (b) the Randall–Selitto test for A-δ- and C-fiber-mediated mechanical hyperalgesia; (c) hindpaw flexion for A-α motor- and A-β non-nociceptive sensory-fiber function; and (d) hindpaw extension for A-α motor-fiber function.^38^^,^^39^^,^^40^^,^^41^
  a. Perform Hargreaves testing.
    ***Note:*** This method quantifies thermal hyperalgesia, predominantly reflecting C-fiber–mediated nociceptive activation, in a controlled and reproducible manner.^39^
    i. Place animals on a plexiglass plate inside an open-bottom plexiglass testing cage and allow them to habituate for 15 min.
    ii. Before the first testing session, habituate animals to the testing cage for 15 min over 2 consecutive days without testing.
    iii. Conduct baseline testing on 2 consecutive days before FUS application and hindpaw incision.
    iv. On each testing day, allow animals to habituate to the testing cage for 15 min before starting the test.
    v. Focus radiant heat on the area 1.5 cm proximal to the heel on the plantar surface of each hindpaw.
    vi. Measure the latency to reflexive withdrawal of the hindlimb using an electronic timer.
    vii. Repeat the test 5 times with a 3-min interval between sessions.
      **Pause point:** Record the withdrawal latency for each trial and calculate the average of the five values for analysis.
  b. Perform Randall–Selitto testing.
    ***Note:*** This method offers a quantifiable measure of mechanical hyperalgesia, evaluating nociceptive withdrawal thresholds in a controlled and reproducible manner, specifically assessing activation of A-δ and C fiber nociceptors.^40^
    i. Handle animals daily for 5–10 min over 2 days to acclimate them to human interaction.
    ii. Habituate animals to the restraint cloth for 2 days without conducting any tests.
    iii. Perform testing on 2 consecutive days before FUS application and hindpaw incision.
    iv. On each testing day, handle animals for 5 min to ensure comfort, then immobilize them using a soft cloth.
    v. Apply the Randall–Selitto device to the proximal portion of the plantar surface of the hindpaw.
    vi. Gradually increase the mechanical force at 5–10 g per second until a withdrawal response or vocalization occurs, limiting the maximum force to 250 g to prevent skin damage.
    vii. Repeat the test 3 times with a 1-min interval between sessions.
      **Pause point:** Record the force at which the withdrawal response occurs and average the three values for analysis.
      **CRITICAL:** Gently press the animal's paw while being mindful of the sutures at the base of the incision.
  c. Perform hindpaw flexion reflex testing.
    ***Note:*** This method evaluates sensorimotor integration, largely reflecting A-β fiber–mediated responses, by assessing hindpaw digit flexion upon contact.^41^
    i. Handle animals daily for 5–10 min over 2 days before the first testing session.
    ii. Conduct testing on 2 consecutive days before FUS application and hindpaw incision.
    iii. Gently grasp the rat around the body and tail and lift it, allowing the plantar surface of the hindpaw to touch a metal grasp bar.
      **Pause point:** Proceed to grade the reflex response once the hindpaw has contacted the grasp bar.
    iv. Grade hindpaw digit extension as 0 (no response), 0.5 (partial response), or 1 (full response).
      ***Note:*** Hindpaw sensation is not required for the startle extension reflex but is necessary for the grasp reflex.
  d. Perform hindpaw extension reflex testing.
    ***Note:*** This method evaluates motor function, primarily reflecting A-α fiber activity, by assessing hindpaw digit extension in a controlled manner.^42^
    i. Handle animals daily for 5–10 min over 2 days before the first testing session.
    ii. Conduct testing on 2 consecutive days before FUS application and hindpaw incision.
    iii. Gently grasp the rat around the body and tail, lift it above the countertop, and observe for hindpaw digit extension.
      **Pause point:** Proceed to grade the reflex response once digit extension has been observed.
    iv. Grade hindpaw digit extension as 0 (no response), 0.5 (partial response), or 1 (full response).
  e. Follow the assessment schedule.
    i. Perform baseline assessments on two consecutive days (days −2 and −1) before FUS application and hindpaw incision.
    ii. Perform an assessment on the day of the procedure (day 0).
    iii. Perform assessments on post-FUS/surgical days (POD) 4, 7, 11, 14, 17, 21, 24, 28, 35, 42, 56, 70, 84, 98, 112, 140, and 168, spanning approximately 24 weeks.
  f. Calculate the daily and baseline values for each animal.
    i. Average the five Hargreaves trials performed on each day to obtain a daily thermal-withdrawal latency.
    ii. Average the three Randall–Selitto trials performed on each day to obtain a daily mechanical-withdrawal threshold.
    iii. Average the values obtained on days −2 and −1 to obtain the baseline value used for subsequent statistical comparisons.
  g. Minimize observer-dependent variability.
    i. Have behavioral assessments performed by three trained experimenters using standardized testing and scoring criteria.
      ***Note:*** Experimenter blinding was not feasible because treatment-group allocation was apparent from the experimental procedures.

## Expected outcomes

This protocol outlines the use of noninvasive FUS for reversibly inhibiting the sciatic nerve to prevent acute pain in a rat incisional pain model. We validated the use of FUS with specific parameters (1.5-MHz continuous wave with burst mode off, i.e., 100% duty cycle; 6.65 MPa focal pressure (peak-to-peak) measured in degassed water; total application time 60 s; ISPPA, spatial-peak pulse-average intensity (equal to the spatial-peak temporal-average intensity, ISPTA, for this continuous-wave exposure) = 1436 W cm^−2^).^2^ Using these parameters, acoustic energy was applied to the sciatic nerve, and reversible inhibition was confirmed through sensory and motor behavioral testing. The FUS+HPI (intervention) group (n = 6) was compared with the HPI only (Disease Control) group (n = 6), with male and female animals randomly allocated between the two groups (FUS+HPI: 3 male and 3 female; HPI only: 3 male and 3 female). In the intervention arm (INT, FUS application followed by hindpaw (HP) incision), animals had significantly attenuated hypersensitivity compared to the HPI only (Disease Control) group. The primary outcomes were between group differences in thermal and mechanical nociceptive thresholds using the modified Hargreaves method and the Randall-Selitto tests, respectively. Secondary outcomes were between group differences in HP extension and flexion. Compared with the HPI Only group, the FUS+HPI group exhibited significantly increased thermal withdrawal thresholds for 4 weeks and significantly increased mechanical withdrawal thresholds for 5 weeks following FUS application. Flexion and extension responses were reduced for 4 and 6 weeks, respectively, with gradual recovery thereafter. In contrast, control animals demonstrated invariant full flexion and extension responses across all time points (Figure S4).^1^ Thus, using the above parameter set, FUS noninvasively induced reversible inhibition of the sciatic nerve, effectively reducing pain behaviors after HP injury.^1^ The reversibility of these deficits suggests FUS is a safe as well as an effective method for peripheral nerve blockade.

With further optimization of acoustic parameters, this approach may allow tunable, variable duration, fiber-specific nerve inhibition tailored to distinct pain phenotypes. Integration with dUS guidance enhances targeting precision and translational applicability. Ultimately, this strategy has the potential to reduce reliance on systemic pharmacologic therapies and invasive peripheral nerve blocks while improving postoperative and post-traumatic pain management outcomes. In a recent study from our laboratory, noninvasive diagnostic ultrasound-guided focused ultrasound (dUS-FUS) was shown to enable selective, reversible inhibition of peripheral nociceptive fibers while preserving motor function and preventing acute pain behaviors.^1^ The anticipated outcomes described here are based on previously published experimental findings demonstrating reversible focused ultrasound-induced peripheral nerve blockade and attenuation of acute pain behaviors.^1^^,^^2^ Representative data from this study are reproduced here to illustrate the expected experimental outcomes.^1^^,^^2^

## Quantification and statistical analysis

For statistical analysis, continuous outcomes from the Randall–Selitto and modified Hargreaves assays were summarized as mean ± 95% confidence interval at each time point. Because each animal was measured repeatedly over time, longitudinal data were analyzed using a two-way repeated-measures (mixed-design) analysis of variance, with treatment group as the between-subject factor and time as the within-subject (repeated-measures) factor, including the group × time interaction. Bonferroni-adjusted post hoc comparisons between groups at each time point were performed where the interaction or main effect was significant. Statistical significance was defined as p < 0.05. Analyses were conducted using GraphPad Prism version 10 (GraphPad Software, San Diego, CA, USA).

An a priori power analysis determined that six animals per study arm would provide 80% power to detect a standardized effect size of 1.25 at a two-sided α of 0.05. Two study arms were included, with six animals per arm (total n = 12); n represents individual animals. Hindpaw flexion and extension outcomes were summarized descriptively without inferential statistical testing because of minimal variability across study arms. No animals or observations were excluded or missing; all animals contributed data at every time point, and no data were imputed. Sex was not included as a factor in the model (3 male and 3 female animals per group); the study was not powered to test sex effects.

## Limitations

This protocol has been developed and validated using adult male and female Sprague Dawley rats for reversible sciatic nerve modulation in an acute hindpaw incision model and has not yet been systematically evaluated in other species, disease models, developmental stages, or anatomical targets. Although the dUS-FUS platform can be adapted to a broad range of superficial and deep tissues, acoustic parameters, targeting strategies, and biological endpoints should be independently optimized for each tissue and experimental application.

Successful implementation of this protocol relies on accurate co-registration of the dUS and FUS transducers, consistent acoustic coupling, and precise image-guided targeting. Small anatomical targets, physiological motion (e.g., respiration and cardiac motion), and limited acoustic windows created by bone or air-filled organs may reduce targeting accuracy. In addition, diagnostic ultrasound inherently involves a trade-off between penetration depth and spatial resolution, whereby lower-frequency imaging improves visualization of deeper tissues at the expense of image resolution, whereas higher-frequency imaging provides superior resolution but is restricted to more superficial targets.

Unlike MRI-guided focused ultrasound systems, the dUS-FUS platform does not provide real-time tissue thermometry during sonication. Consequently, acoustic dose must be established through prior hydrophone calibration and parameter optimization rather than direct measurement of tissue temperature. Furthermore, the −3 dB focal zone represents a gradual reduction in acoustic intensity rather than a sharply defined treatment boundary. Combined with differences in tissue acoustic properties, including absorption, attenuation, and perfusion, this may produce variability in energy deposition and biological responses and may result in partial exposure of adjacent tissues. Careful calibration and validation are therefore recommended before applying this protocol to new tissues or experimental models.

The platform also requires specialized instrumentation, precise transducer alignment, degassed water coupling, and careful animal positioning, which may reduce experimental throughput compared with simpler pharmacological or surgical approaches. Finally, acoustic exposure parameters optimized in rodents do not scale directly to larger animal models or humans because of differences in anatomy, tissue dimensions, and acoustic propagation. The use of general anesthesia may also influence physiological and behavioral outcomes and should be considered when interpreting experimental results.

## Troubleshooting

### Problem 1

Vacuum tank leakage (step 16a). Possible cause: Loose ends, improperly closed knobs, or loosened rubber seals.

### Potential solution

Inspect the rubber seal, ensure all knobs are tightly closed, or reseal with silicone gel for proper tightening.

### Problem 2

Air bubbles observed on the FUS transducer (step 17). Possible cause: Trapped water in the tank or inadequate degassing of the water before use.

### Potential solution

Remove bubbles using a syringe, gently move the FUS up and down, use degassed water, immerse slowly to minimize turbulence, and allow settling time.

### Problem 3

Voltage signal is too low or noisy (step 17). Possible cause: Improper coupling, air bubbles, misalignment, faulty cables, electrical interference, damaged hydrophone, contaminated water, insufficient power, or incorrect signal settings.

### Potential solution

Ensure proper coupling, remove air bubbles, align accurately, check cables, shield from noise, use degassed water, and verify system settings.

### Problem 4

FUS transducer temperature-probe data logging failure (step 18d). Possible cause: Improper cable connections, uninitiated logging, incorrect folder selection, or data saved to an inaccessible default location.

### Potential solution

Ensure proper cable connections, initiate logging, select the correct folder, and verify the save location.

### Problem 5

dUS image appears too dim, unclear, or hindered by tissue interference (step 18e). Possible cause: Poor contact, incorrect settings, dense tissue, probe-tissue contact, weak signal, or dirty degassed water.

### Potential solution

Position the probe correctly, adjust the settings, replace dirty water, prepare the tissue properly, dip it in clean water for imaging, and maintain a distance from the tissue.

### Problem 6

Animal fixation in small tanks (step 18b). Possible cause: Improper animal weight (too heavy or too light) or low bed height disrupts dUS-FUS alignment, as incorrect bed adjustments or unsuitable animal size hinder proper setup positioning.

### Potential solution

Place a platform under small animals, avoid overweight subjects, ensure proper weight for water immersion, and stabilize the dUS-FUS setup.

### Problem 7

FUS transducer lens temperature rises excessively during animal tissue application (steps 18d and 18e). Possible cause: The coolant system may be off or connections disconnected.

### Potential solution

Check cable connections and ensure the coolant system is on during FUS application to prevent abrupt temperature rise. Stop the sonication immediately (turn off the FG output) if the logged transducer temperature exceeds 40 °C, and resume only after the cause has been corrected.

### Problem 8

Skin burns or lesion after FUS application to animal tissue (step 18e). Possible cause: thermal damage, excessive acoustic intensity, insufficient cooling, uneven tissue surface, hair follicles, poor coupling medium, and air gaps between the transducer and tissue.

### Potential solution

Stop the sonication immediately by turning off the FG output. Complete all of the following checks before any further sonication:

1. Inspect the skin at the target site (blanching, erythema, blistering, or lesion), record the finding, and consult veterinary staff according to institutional guidelines if skin injury is present.
2. Inspect the transducer face and the acoustic path for air bubbles or gaps and remove them with a syringe; confirm that the coolant is circulating and that the logged transducer temperature is within the safety cutoff.
3. Confirm that the target skin surface is free of hair and has been cleaned (e.g., with 70% ethanol), that the coupling is even, and that the transducers do not touch the skin.
4. Confirm that the FG settings (Step 18e) and the RF amplifier output match the parameters described in this protocol.
5. Confirm that the co-registration and the focus mark on the imaging screen are unchanged (Steps 14 and 18e).

Do not move the transducers and the animal closer together and do not change the acoustic parameters ad hoc. The focus is fixed at the co-registered depth (∼16.5 mm), so changing the distance or the parameters alters the delivered dose and requires re-calibration with the needle hydrophone (Steps 13, 14, and 17) before further use. Resume sonication only after the cause has been identified and corrected.

### Problem 9

Animals respond to a pinch or reflexes during surgery (step 19b). Possible cause: anesthesia may be inappropriate for the animal, not activated during maintenance, or require adjustment due to insufficient isoflurane levels in the setup.

### Potential solution

Ensure anesthesia is properly adjusted to the animal’s body weight, adequately filled, and turned on for effective maintenance.

## Resource availability

### Lead contact

Further information and requests for resources should be directed to and will be fulfilled by the lead contact, Anderson T.A. (tanders0@stanford.edu).

### Technical contact

Technical questions regarding the construction, calibration, and operation of the dUS-FUS platform should be directed to Anderson T.A. (tanders0@stanford.edu) and Sangwan N. (snamrata@stanford.edu).

### Materials availability

This study did not generate new unique reagents. The associated 3D design and fabrication files, as well as additional information regarding the fabrication, assembly, and use of the custom-built components, will be available from the lead contact upon reasonable request.

### Data and code availability

• All data supporting the findings of this study are included in the article and its supplemental information. Additional raw data will be available from the lead contact upon reasonable request.
• The custom MATLAB scripts used to control the function generator are provided with this protocol as a supplemental data file (Data S1). No other custom code was generated in this study; statistical analyses were performed in GraphPad Prism version 10. Any additional information required to reanalyze the data reported in this paper is available from the lead contact upon request.

## Acknowledgments

A.T.A. acknowledges an award from the National Institutes of Health (NIH; R18EB035005) and support from the Stanford-Coulter Translational Research Grant Program, Stanford University, California, USA. The author also acknowledges SONELE Inc. for technical support and system-related contributions. The authors sincerely thank Dr. Michael Klukinov and Dr. David C. Yeomans for their support in establishing the acute incisional pain model. Schematics were created using BioRender.com. This research was supported by the National Institutes of Health (NIH) under Award No. R18EB035005 and a Stanford-Coulter Translational Research Grant. The content is solely the responsibility of the authors and does not necessarily represent the official views of the NIH or any other funding agency.

## Author contributions

A.T.A. and S.N. conceived the study and designed the research. A.T.A. and S.N. characterized and standardized the dUS-FUS system. N.C.C. and T.Z. performed the manufacturing, fabrication, and optimization. S.N. and A.T.A. conducted the experiment. S.N. drafted the manuscript, and A.T.A. reviewed and edited the manuscript.

## Declaration of interests

C.N.C. and T.Z. are employees of SONELE Inc. (Markham, Ontario, Canada), which designed and manufactured the FUS transducer, hydrophone and small-animal tanks, black-box control module, and related hardware used in this protocol (see key resources table). SONELE manufactures and supplies this hardware. SONELE had no role in the design of the animal experiments, data collection, analysis, or the decision to publish.

## Supplemental information

Document S1. Figures S1–S4

Data S1. Custom MATLAB script for function generator control
