# Supplementary material for: Image-guided focused ultrasound for non-invasive modulation of superficial and deep tissues
Source: STAR Protoc. 2026 Oct 19;7(4):104900. doi: 10.1016/j.xpro.2026.104900 (PMC13695158; doi:10.1016/j.xpro.2026.104900)
Supplement: Supplementary file 1 [file mmc1.pdf]

Image-guided focused ultrasound for non-invasive modulation of superficial and deep tissues

[1]Sangwan N, [2]Chaggares N C, [2]Torbatian Z, [1]Anderson TA
[1]Department of Anesthesiology, Perioperative and Pain Medicine, School of Medicine, Stanford University, CA, USA.
[2]SONELE Inc., Markham, Ontario, Canada

Author Information:
Dr. Namrata Sangwan, PhD: Department of Anesthesiology, Perioperative and Pain Medicine, School of Medicine, Stanford University, CA, USA. Email- snamrata@stanford.edu
Chris N Chaggares, MASc: SONELE Inc., Markham, Ontario, Canada. Email- nchrisc@sonele.com
Zahra Torbatian, PhD: SONELE Inc., Markham, Ontario, Canada. Email- zahrat@sonele.com
Dr. Thomas Anthony Anderson, PhD, MD: Department of Anesthesiology, Perioperative and Pain Medicine, School of Medicine, Stanford University, CA, USA. Email- tanders0@stanford.edu

Lead contact: Thomas Anthony Anderson, PhD, MD (tanders0@stanford.edu)
Technical contact: Thomas Anthony Anderson, PhD, MD (tanders0@stanford.edu) and Namrata Sangwan, PhD (snamrata@stanford.edu)
***Correspondence:** Thomas Anthony Anderson (tanders0@stanford.edu)

Address correspondence to:
Thomas Anthony Anderson, PhD, MD
Department of Anesthesiology, Perioperative and Pain Medicine
Stanford University School of Medicine
Palo Alto, CA, USA
Phone: 650-725-5742
Email: tanders0@stanford.edu
ORCID: http://orcid.org/0000-0002-6004-6102

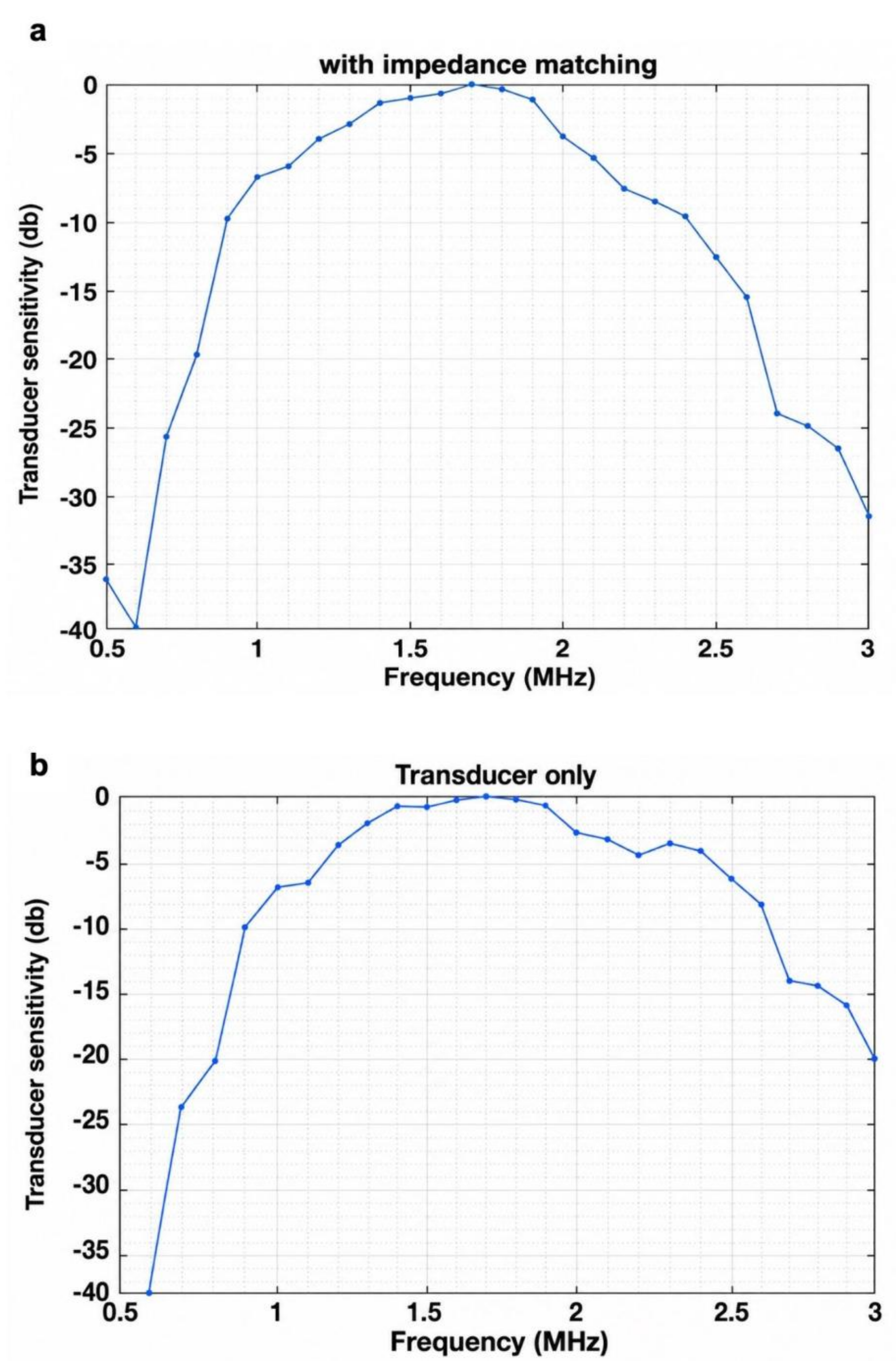

Figure S1 | Frequency response of the 1.5-MHz FUS transducer (Step 12b). (1) Transducer RMS pressure sensitivity measured across frequency with the 1.5-MHz impedance matching circuit connected. **(2)** Transducer RMS pressure sensitivity measured across frequency without the impedance matching circuit. Measurements were acquired at the acoustic focus using a calibrated hydrophone. The −6 dB transmit bandwidth was 1.08–2.13 MHz (65%) with impedance matching and 1.10–2.51 MHz (78%) without impedance matching.

sig gen-->RF amp--> impedance mating-->Transducer

rho_c 1500000

attn used for detaing MI: 0.3db/cm/MHz--->0.76 db at 17.4--->1.09

Eqv sig gen for customer setting	Sig Gen (mV)	RF gain (customer	Vpkpk-measured on scope with ATTN (50 ohm)	Max_P (Mpa)	Min_P (MPa)	RMS-P (Mpa)	peak-topeak pressure	Prms^2/rho_c	Isppa	Ispta	MI-dr	Estimated RFB-CW, 5sec	Measured thermal rise in phantom using themocouple-
25	25.9	378	9.79	0.341	0.319	0.235	0.66	36816.66667	3.68	1.84	0.24	0.16	
50	49.44	378	18.69	0.675	0.616	0.462	1.29	142296	14.23	7.11	0.46	0.57	
100	98.89	378	37.38	1.42	1.2	0.932	2.62	579082.6667	57.91	28.95	0.90	2.26	
125	124.79	378	47.17	1.826	1.47	1.146	3.30	875544	87.55	43.78	1.10	3.61	
138	137.97	378	52.154	2.04	1.6	1.286	3.64	1102530.667	110.25	55.13	1.20	4.41	~20 deg C rise in 20 sec
150	150.69	378	56.96	2.257	1.72	1.39	3.98	1288066.667	128.81	64.40	1.29	5.26	
180	182	378	68.797	2.76	2.019	1.686	4.78	1895064	189.51	94.75	1.51	7.67	
200	200.13	378	75.65	3.08	2.197	1.848	5.28	2276736	227.67	113.84	1.65	9.27	
250	249.58	378	94.34	4.038	2.61	2.277	6.65	3456486	345.65	172.82	1.96	14.42	
280	280.19	378	105.91	4.63	2.87	2.56	7.50	4361811.86	436.18	218.09	2.15	18.18	
300	299.02	378	113.03	4.94	3.06	2.73	8.01	4967986.97	496.80	248.40	2.29	20.71	
330	329.63	378	124.6	5.45	3.38	3.01	8.82	6037109.84	603.71	301.86	2.53	25.16	~50 deg C rise in 5 sec +mark in HIFU phantom after 35 sec

Figure S2 | Acoustic output characterization of the 1.5-MHz FUS transducer. Quality-control measurements showing the relationship between transmit voltage and acoustic output parameters, including peak pressure, RMS pressure, acoustic intensity, and mechanical index, obtained using a calibrated hydrophone and 1.5-MHz impedance matching circuit.

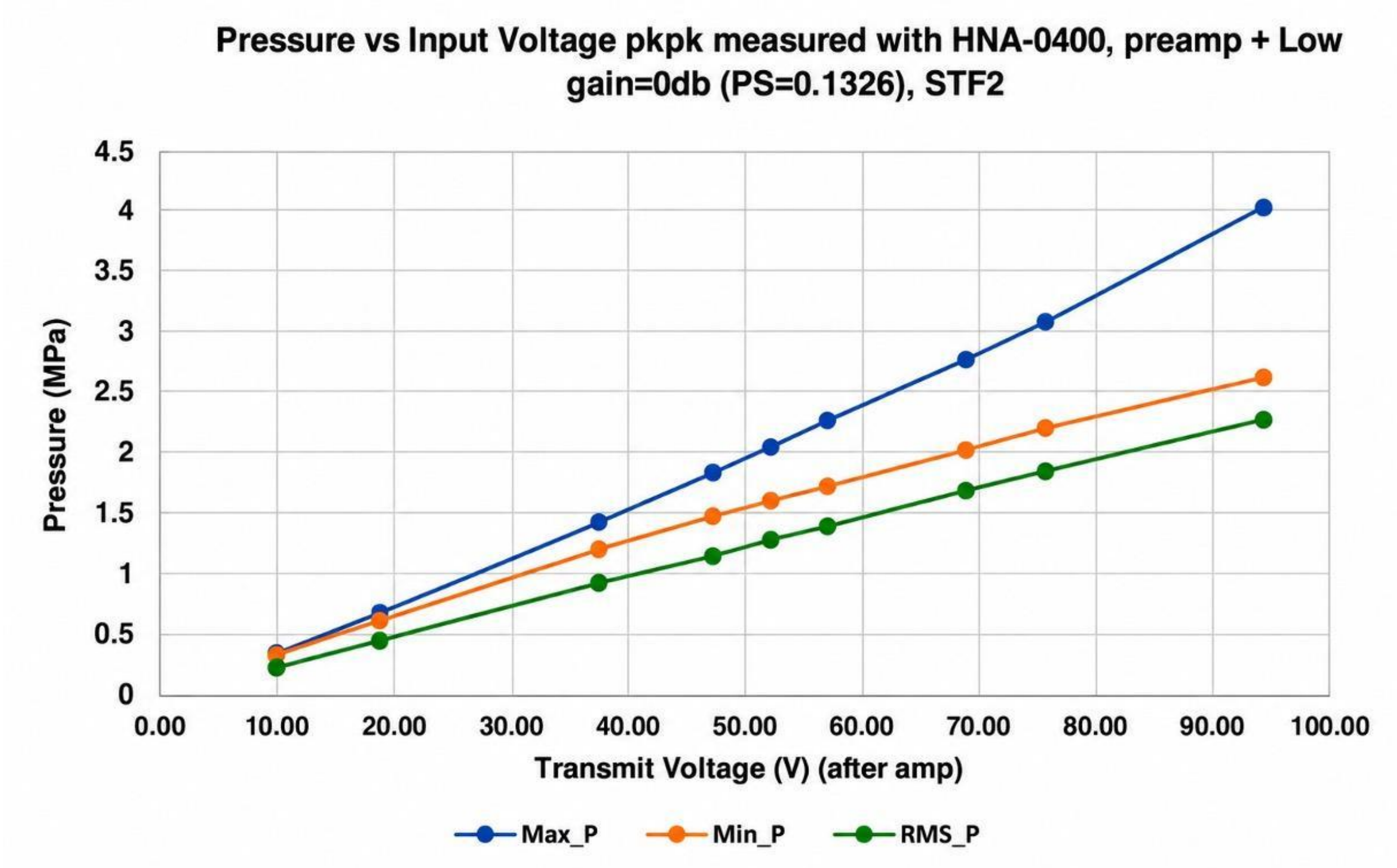

Figure S3 | Pressure–voltage calibration of the 1.5-MHz FUS transducer. Maximum pressure (Max_P), minimum pressure (Min_P), and root mean square pressure (RMS_P) measured at the acoustic focus as a function of transmit voltage using a calibrated hydrophone.

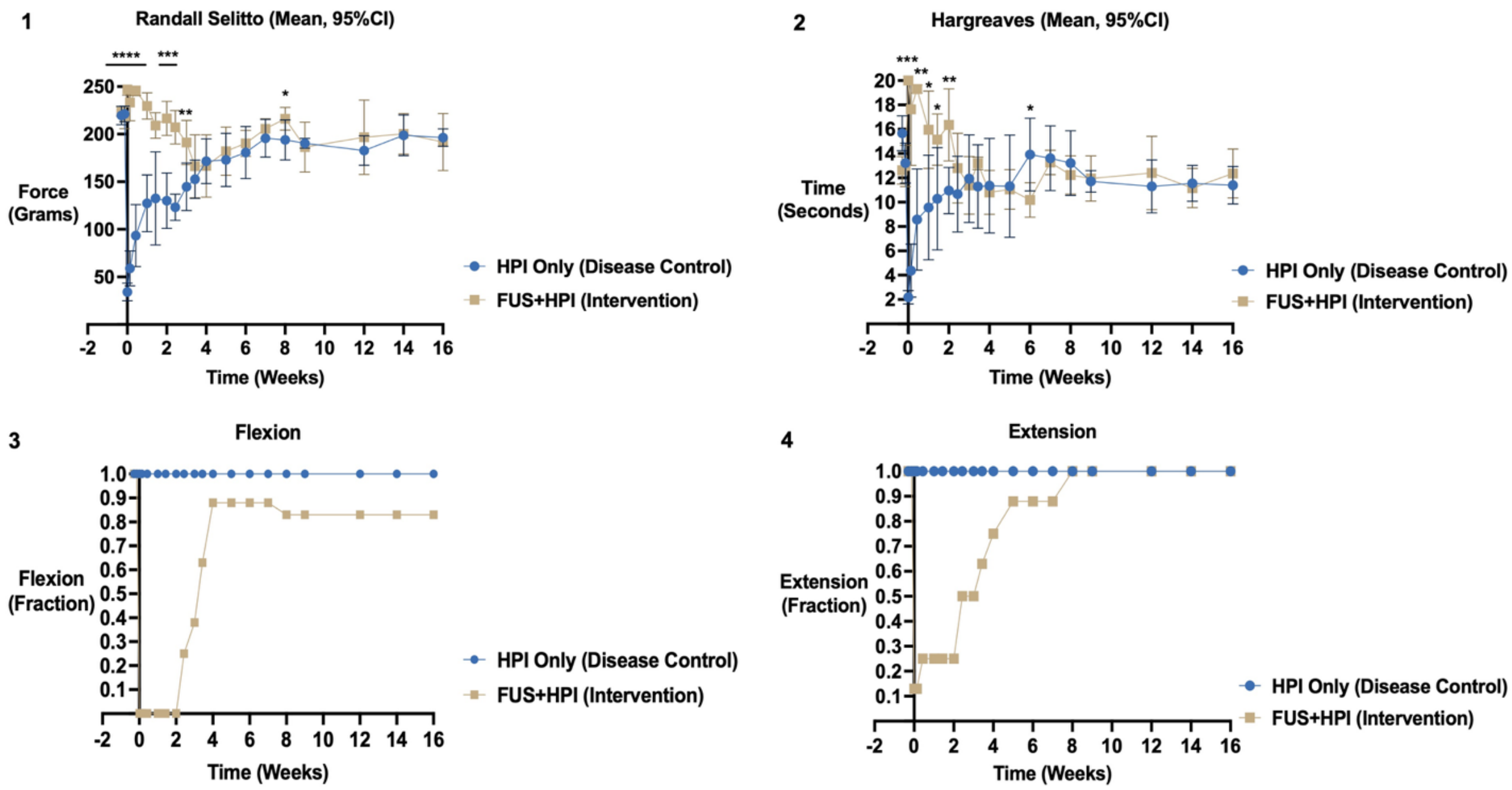

Figure S4 | Behavioral outcomes in the Intervention and Control arms (Step 20). (1) Mechanical withdrawal threshold (Randall Selitto test); **(2)** thermal withdrawal latency (modified Hargreaves test); **(3)** hindpaw (HP) flexion; (4) hindpaw (HP) extension. Panels 1 and 2: Data are presented as mean ± 95% confidence interval (CI). n = 6 animals per group (3 male and 3 female). Group differences over time were assessed using a two-way repeated-measures (mixed-design) ANOVA with treatment group as the between-subject factor and time as the within-subject factor, followed by Bonferroni-adjusted post hoc comparisons between groups at each time point. Asterisks denote statistical significance: *$p < .05$, **$p < .01$, ***$p < .001$, ****$p < .0001$. Panels 3 and 4: Data are presented descriptively, without inferential statistical testing, as control animals exhibited no variance at these time points. FUS, focused ultrasound; HP, hindpaw; INT, intervention arm.
